# Are Nutraceuticals Beneficial in Chronic Kidney Disease?

**DOI:** 10.3390/pharmaceutics13020231

**Published:** 2021-02-06

**Authors:** Jacek Rysz, Beata Franczyk, Krzysztof Kujawski, Izabela Sacewicz-Hofman, Aleksanda Ciałkowska-Rysz, Anna Gluba-Brzózka

**Affiliations:** 1 Department of Nephrology, Hypertension and Family Medicine, Medical University of Lodz, 90-549 Lodz, Poland; jacek.rysz@umed.lodz.pl (J.R.); bfranczyk-skora@wp.pl (B.F.); kkujawski@poczta.fm (K.K.); 2 Department of Molecular Cell Mechanisms, Medical University of Lodz, 90-549 Lodz, Poland; izabela.sacewicz-hofman@umed.lodz.pl; 3 Palliative Medicine Unit, Department of Oncology, Medical University of Lodz, 90-549 Lodz, Poland; olarysz@rmed.pl

**Keywords:** chronic kidney disease, nutraceutical, resveratrol, curcumin

## Abstract

Chronic kidney disease (CKD) is a worldwide health problem in which prevalence is constantly rising. The pathophysiology of CKD is complicated and has not been fully resolved. However, elevated oxidative stress is considered to play a vital role in the development of this disease. CKD is also thought to be an inflammatory disorder in which uremic toxins participate in the development of the inflammatory milieu. A healthy, balanced diet supports the maintenance of a good health status as it helps to reduce the risk of the development of chronic diseases, including chronic kidney disease, diabetes mellitus, and hypertension. Numerous studies have demonstrated that functional molecules and nutrients, including fatty acids and fiber as well as nutraceuticals such as curcumin, steviol glycosides, and resveratrol not only exert beneficial effects on pro-inflammatory and anti-inflammatory pathways but also on gut mucosa. Nutraceuticals have attracted great interest recently due to their potential favorable physiological effects on the human body and their safety. This review presents some nutraceuticals in which consumption could exert a beneficial impact on the development and progression of renal disease as well cardiovascular disease.

## 1. Introduction

Chronic kidney disease (CKD) is a worldwide health problem in which occurrence is constantly rising. According to estimations, its prevalence amounts to 8–16% of populations worldwide [1,2]. Patients with CKD have an extremely high risk of cardiovascular disease (CVD) and they are even 10 times more likely to die from cardiovascular disease before reaching the CKD stage requiring dialysis or kidney transplantation compared to age-matched controls [1,2]. This disease is characterized by progressive loss of kidney function, resulting in the diminished ability of the body to eliminate soluble waste and consequent accumulation of “uremic toxins” [3,4,5]. According to K/DOQI (Kidney Disease Outcomes Quality Initiative), CKD is defined as kidney damage confirmed by structural or functional abnormalities of the kidney or glomerular filtration rate (GFR) <60 mL/min/1.73 m^2^ for 3 months or more, irrespective of cause [1]. The pathophysiology of CKD is complicated and has not been fully resolved. However, elevated oxidative stress is considered to play a vital role in the development of this disease [6]. CKD is also thought to be an inflammatory disorder in which uremic toxins are involved in the development of the inflammatory milieu [3,4,5]. Chronic kidney disease is also characterized by a diminished antioxidant capacity. The presence of the inflammatory state is associated with the recruitment of immune system cells (macrophages and leucocytes) to the site of the injured kidney [7]. The resulting “respiratory burst” is associated with the overproduction of reactive oxygen species (ROS) resulting in enhanced oxidative stress, monocyte adhesion, endothelial dysfunction, smooth muscle cell proliferation, vascular calcification, and accelerated atherosclerosis [8,9]. The inflammatory chain reaction in the kidney leads to its further damage [10]. Among numerous pro-inflammatory cytokines, tumor necrosis factor-α (TNFα), interleukin 1β (IL-1β), and interleukin-6 (IL-6) seem to play the most important role in CKD [9]. In turn, growth factors including transforming growth factor β (TGFβ), vascular endothelial growth factor (VEGF), and platelet-derived growth factor (PDGF) are involved in the glomerular cell proliferation and glomerular extracellular matrix expansion, which contribute to renal failure [3,11]. The progression of chronic kidney disease leads to endothelial dysfunction as the result of the presence of the eNOS endogenous inhibitor-asymmetric dimethylarginine (ADMA), decreased NO bioavailability, and/or insufficient synthesis of NO. These disturbances further result in hypertension and atherosclerosis as well as the development of several alterations in the innate and adaptive immune systems, which increases the risk of atherogenesis and vascular disease [7,12,13,14]. Obese/diabetic patients with reduced eGFR have high levels of reactive oxygen species and decreased levels of antioxidants, which confirm the hypothesis about the crosstalk between oxidative stress, obesity/diabetes/diabesity, and CKD.

A healthy, balanced diet supports the maintenance of a good health status as it helps to reduce the risk of the development of chronic diseases, including chronic kidney disease, diabetes mellitus, and hypertension [15]. Healthy eating can decrease the workload on the diseased kidneys and enable the regulation of residual kidney function, the control of wastes build-up in the body, and the prevention of infection and muscle loss [16,17]. Vast evidence indicates that gut microbiota balance can prevent renal function decline [18]. The control of low-grade chronic inflammation is also of key importance for renal damage prevention [15]. Numerous studies have demonstrated that functional molecules and nutrients, including fatty acids and fiber as well as nutraceuticals, such as curcumin, steviol glycosides, and resveratrol not only exert beneficial effects on pro-inflammatory and anti-inflammatory pathways but also on gut mucosa [3,19]. The consumption of products that are rich sources of antioxidants has been demonstrated to be associated with significant benefits related to the reduction of oxidative stress in different health conditions [6].

The selection of articles for this literature review was based on PubMed search (terms: “nutraceuticals” + “CKD” were applied) and, due to the fact that there are so many nutraceuticals, we decided to choose those which are most frequently mentioned. In addition, we concentrated on those studies that were performed on large groups or which results were, in our opinion, particularly interesting.

## 2. Nutraceuticals

Nutraceuticals have recently attracted great interest due to their potential favorable physiological effects on the human body and their safety. The first definition of “nutraceuticals” was formed in 1989 by Dr Stephen L. De Felice, a founder and chairman of the Foundation for Innovations in Medicine [20,21]. According to the definition, nutraceuticals are any natural non-toxic food products, extracts, or food derivatives containing bioactive chemical substances, such as herbs, vitamins, amino acids, minerals, and enzymes with scientifically proven beneficial properties in the field of disease prevention and treatment [22,23]. These substances have been suggested to provide protection against chronic diseases including renal disease diabetes, neurological and gastrointestinal disorders, and cancers [24]. Moreover, they can be used to delay the ageing process and increase life expectancy. Some nutraceuticals are included in foods and are also available in the form of tablets, capsules, or powder [24]. The use of nutraceutical varies worldwide. It has been estimated that more than 50% of adults in some Western countries regularly consume them [23,25]. The popularity of nutraceuticals is also associated with the fact that many of them are easily available, cheap, and in low-doses. They primarily do not cause unpleasant side-effects [26]. However, there are also drawbacks of nutraceuticals, which should be mentioned. According to some studies, these compounds may have poor bioavailability. They are easily eliminated from the body and, thus, they cannot provide sufficient medicinal benefit [27]. Moreover, due to the lack of strong evidence and final recommendations, in case of many products, there are no clear data on their safety and effectiveness, possible side effects, interactions with prescribed medicines or on their impact on the existing, medical conditions [28]. Some nutraceuticals may have their own inherent toxicity and may cause adverse interactions with drugs prescribed for renal disease [24].

The use of nutraceuticals in the prevention of renal dysfunction is a very tempting option. A number of nutraceutical products are being promoted as effectively supporting, protecting, or even healing renal injuries. However, there are no hard results advocating this thesis [24]. It is believed that bioactive compounds of nutraceuticals play a vital role in suppressing macro-mechanistic processes in patients with chronic kidney disease and they could reduce the inflammation, oxidative stress, and sympathetic overactivation, promote renal blood flow and glomerular filtration rate, upregulate antioxidant properties, raise NO bioavailability as well as inhibit interstitial fibrosis, and stimulate tubular regeneration [15]. Strategies reducing ROS levels and enhancing the antioxidant capacity of the body are also used in the management of obesity and diabetes in which the latter frequently impairs renal function [29]. Some studies provided evidence that nutraceuticals could improve vascular-related outcomes in animal models and healthy people. However, hardly any data show the beneficial effects on vascular health in patients with CKD [12,30]. Therefore, it seems that the development of nutraceutical therapies and the understanding of mechanisms via which they may counteract vascular disturbances in CKD may be of high importance [12].

Numerous animal-based studies have been performed to find the “golden compound” in the field of nephrology. Al-Okbi et al. [31] assessed protective effects of extracts prepared from avocado, walnut, flaxseed, and *Eruca sativa* seeds in a rat model of kidney dysfunction induced by intraperitoneal cisplatin. In their study, the pre-treatment with different nutraceuticals was associated with a strong protection toward kidney dysfunction mirrored by a considerable decrease in the level of plasma creatinine and urea as well as the increase in plasma albumin, plasma total protein, and creatinine clearance. The administration of studied nutraceuticals resulted in the enhancement of total antioxidant capacity and catalase activity, the decrease in free radicals, and subsequent improvement of kidney function due to the limit of damage and an inflammatory process. The observed histopathological changes confirmed the effects noticed in that study. A nutraceutical-related drop in free radicals’ levels also caused the reduction of chromosomal aberrations. According to authors, a renal protective effect of analyzed nutraceuticals might be related to the presence of phenolic compounds, polyunsaturated fatty acids, and phytosterols. In addition, in other studies, the antioxidant and anti-inflammatory activity of phenolic compounds have been observed [32,33]. Phytosterols have been demonstrated to exert antioxidant and anti-inflammatory effects, while polyunsaturated fatty acids were found to have anti-inflammatory activity [34,35]. In turn, Almomen et al. [36] analyzed the beneficial effect of whole grape powder (WGP) in rats with CKD associated with a metabolic syndrome. Their study demonstrated lower proteinuria and urination as well as the reduction of common features present in early stages of diabetes-related CKD, such as mesangial expansion, glomerular atrophy tubular injury, and protein cast formation in the WGP-fed rats. Renal protection in rats on the WGP diet seems to be associated with the up-regulation of antioxidants: 24-dehydrocholesterol reductase (Dhcr24), glutathione S-transferase kappa 1 (Gstk1), peroxiredoxin 2 (Prdx2), superoxide dismutase 2 (Sod2), glutathione peroxidase 1 and 4 (Gpx1 and Gpx4), and down-regulation of thioredoxin interacting protein (Txnip) (for ROS production) in the kidneys. However, the use of some nutraceuticals may also be associated with adverse effects. Luciano RL [37] described a case of hemodynamically mediated acute kidney injury and hyperkalemia in patients with chronic kidney disease following daily consumption of cherry concentrate. The author suggested that the compounds comprised in cherries inhibited cyclooxygenase in a mechanism mimicking the action of nonsteroidal anti-inflammatory medications [37].

### 2.1. Polyphenols

Vast epidemiological evidence confirms that a diet abundant in polyphenol-rich compounds such as fruits, vegetables, spices, nuts, cocoa, red wine, and tea can protect against the development of several chronic diseases, including cardiovascular disease [12,38,39]. Results from studies of healthy and clinical populations have indicated that polyphenols improve vascular/cardiovascular functioning via the increase in endothelial function, and decrease of arterial stiffness and blood pressure as well as the inhibition of platelet aggregation [12,40,41,42,43]. Moreover, they were shown to attenuate oxidative stress and modulate cellular signaling pathways including VEGF-mediated angiogenesis, nitric oxide (NO) signaling, endoplasmic reticulum stress, and the Nrf-2-antioxidant pathway [12,15,44,45].

Polyphenols affect pathways involved in the induction of several antioxidant enzymes including glutathione (GSH)-S-transferase (GST) and nicotinamide adenine dinucleotide phosphate (NADPH): quinone oxidoreductase 1 [45,46]. Finally, they exert anti-inflammatory actions by modulating transcriptional networks and/or signaling cascades involved in the regulation of gene expression and the hindering of inflammatory mediators (TNF-α, NF-κB, IL-6, and C-reactive protein) [12,47].

It has been indicated that polyphenols could considerably diminish circulating uric acid and exert a protective effect against vascular oxidative stress, possibly via the inhibition of xanthine oxidase, which is required to produce uric acid but also through their actions as ROS scavengers [48,49,50]. Due to the fact that toxic uremic metabolites were shown to originate not only from intermediary metabolism but also from the gut, a direct influence of diet and bioactive nutrients on microbial metabolism have gained great interest [51]. The consumption of active compounds including polyphenols can modify the composition and metabolism of the microbiota, act as a modulator of transcription factors involved in inflammation and oxidative stress, exert influence on the epigenome by altering one-carbon metabolism, and mitigate mitochondrial dysfunction. Numerous studies have suggested that chronic kidney disease exerts an important impact on the affected individuals with an altered gut flora [24]. It has been shown that gut permeability is involved in the onset and the progression of chronic inflammation as the result of the exposure of gut-associated lymphoid tissue (GALT) to luminal antigens [52]. Gut dysbiosis, which is associated with inflammation and increased cardiovascular risk, has been found to be a common feature in CKD [51]. Intake of prebiotics, probiotics, polyphenols, sugars, and proteins could alter the diversity of the gut microbiota and the generation of uraemic toxins [51].

Below, we will review the beneficial effects of polyphenols: curcumin, resveratrol (RSV), and other active compounds.

#### 2.1.1. Curcumin

Curcumin is a biologically active polyphenolic compound, which exerts anti-inflammatory, and anti-cancer effects [53]. It can be found in turmeric, which is a spice derived from the rhizomes of the plant *Curcuma longa* [15,54]. Curcumin has been tested in clinical trials for various diseases including Alzheimer’s disease, cancer, and ulcerative colitis since numerous preceding animal studies indicated that curcumin could modulate growth and transcription factors, signaling molecules, enzymes, receptors, cytokines, micro-RNA (miRNA), and reactive oxygen species, positively affecting inflammation and oxidative stress [3,55,56,57,58]. Actions of curcumin are exerted on a renal, but also systemic level [15]. The results of in vivo and human studies have suggested that curcumin use may prove beneficial in patients with CKD [59,60], renal transplantation [55,61], acute kidney injury (AKI) [62], and renal cell carcinoma [53]. However, randomized clinical trials are required to confirm the observed effects.

Curcumin and fiber can either directly or indirectly modulate the integrity of the gut barrier [15]. The activity of curcumin at the intestinal level is associated with its systemic effects, including the development of CKD and atherosclerosis. Some articles have demonstrated that curcumin is capable of preserving the intestinal barrier integrity, limiting in consequence of a systemic inflammation [15]. Intestinal lumen lipopolysaccharide (LPS) was shown to alter gut permeability and to expose gut-associated lymphoid tissue to mucosal antigens, thus, increasing intestinal permeability and launching the cascade of inflammatory events, such as the activation of macrophages and their infiltration within the renal tissues as well as enhanced secretion of pro-inflammatory cytokines and chemokines [3,15,63]. The protection of mucosal barrier integrity is associated with the activity of intestinal alkaline phosphatase (IAP), which detoxifies luminal LPS [64]. Oral intake of curcumin has been shown to promote increased IAP activity, which is commonly reduced in CKD patients [7].

#### 2.1.2. Animal Studies

In the study of animal models of CKD (nephrectomized rats), oral administration of curcumin restored systolic blood pressure and ejection fraction, reduced left ventricle dimension at end-systole (LVSd), and decreased interventricular and rear wall thickening ameliorating cardiac function [59]. Moreover, it diminished ROS production, gelatinase activity of metalloproteinases, and the expression of an extracellular matrix remodeling enzyme—metalloproteinase 2 (MMP-2). Curcumin also improved mitochondrial integrity and functionality, but it did not affect renal function. Therefore, it seems that curcumin due to its potential to attenuate oxidative stress-related events, such as cardiac remodeling, mitochondrial dysfunction, and cell death, could be possibly used in the therapy of heart disease in patients with CKD [59]. Curcumin have been shown to improve cardiovascular condition in animals with CKD. Cardiac hypertrophy that develops as a result of renal failure can lead to heart failure. The study of adult Sprague-Dawley rats, which underwent nephrectomy demonstrated that curcumin attenuated cardiac hypertrophy and remodeling via the deactivation of multiple hypertrophic signaling pathways, including the ERK/mTOR (extracellular signal-regulated kinase/ mammalian target of rapamycin) pathway [65]. Mimicking the scenario for renal disease in humans, Ghosh at al. [66] treated 5/6 nephrectomized (Nx) rats with already established proteinuria (after 6 weeks) with curcumin or enalapril. They demonstrated that curcumin was as effective as enalapril in the reduction of inflammatory cytokines TNFα and IL-1β levels and it efficiently abated both proteinuria and kidney injury as manifested by glomerulosclerosis and tubulointerstitial injury [66]. Curcumin administration considerably reduced macrophage infiltration and limited cytokine-mediated elevation of kidney phospholipase and cyclooxygenase, thus, inhibiting the formation of inflammatory eicosanoids adversely affecting kidney function [66]. Owing to its multifaceted actions, curcumin could also be involved in the prevention of renal function decline. Curcumin was demonstrated to prevent renal dysfunction by ameliorating renal blood flow and the total antioxidant capacity in rats after five-sixths nephrectomy (5/6NX) [67]. Authors observed that curcumin pre-treatment reduced early 5/6NX-induced disturbed mitochondrial dynamics, bioenergetics, and oxidative stress, which may be related with the protection of renal function. Jacob et al. [68] using an animal model of immune complex-mediated complement-dependent glomerulonephritis, demonstrated that curcumin considerably improved renal function, diminished glomerulonephritis, and lowered IgG and C9 deposits indicating reduced complement activation. Moreover, it decreased mRNA expression of inflammatory proteins, monocyte chemoattractant protein-1, and transforming growth factor-β as well as matrix proteins, fibronectin, laminin, and collagen [68].

The expression of pro-inflammatory cytokines and adhesion molecules (major mediators of inflammation) is stimulated by reactive oxygen species [69]. TNFα has been demonstrated to down-regulate peroxisome proliferator-activated receptor γ (PPARγ) by abolishing its strong, anti-inflammatory effects [70,71]. However, the administration of curcumin in rats with CKD-related up-regulated expression of PPARγ abrogated TNFα-mediated down-regulation of PPARγ [71]. Moreover, Aggarwal [57] reported that curcumin efficiently antagonized the activation of NF-κB. Shin et al. [72] have demonstrated that long-term administration of curcumin protected against atherosclerosis by decreasing plasma and hepatic cholesterol and also by diminishing β-hydroxy β-methylglutaryl-CoA (HMG-CoA) reductase. This nutraceutical inhibited the transcription of HMG-CoA reductase independent of acetyl-CoA acetyltransferase (ACAT1 and ACAT2) expression. It also upregulated the expression of hepatic PPARα and liver X receptor α (LXRα). Furthermore, Liu et al. [73] suggested that curcumin-related amelioration of atherosclerosis was associated with the stimulation of an ATP-binding cassette transporter (ABCA1), scavenger receptor, class B type 1 (SR-B1), LXR expression, and the promotion of cholesterol efflux. The study of low-density lipoprotein (LDL) receptor deficient (Ldlr(-/-)) mice fed with a low fat (LF) or high fat (HF) diet supplemented with curcumin for 16 weeks revealed that curcumin dose-dependently reduced uptake of oxLDL in THP-1 cells [74]. Curcumin medium doses (500–1000 mg/kg diet) was effective at decreasing fatty streak formation, suppressing aortic expression of IL-6 in the descending aorta, and reducing blood levels of several inflammatory cytokines. However, it turned out that, at a higher dose (HF + HC, 1500 mg/kg diet), curcumin might exert adverse effects on some of these parameters. Hasan et al. [74] suggested that mechanisms via a low dose curcumin modulated atherogenesis involved in the suppression of aP2 and CD36 expression in macrophages (vital factors in atherogenesis). Another study performed on the same animal model indicated that daily administration of 100 mg/kg curcumin resulted in the reduction of the inflammatory markers but it also improved atherosclerosis and glucose intolerance [75]. Curcumin has also been suggested to exert a urate-lowering effect [49]. The study of rats subjected to chemically induced oxidative stress and treated with curcumin demonstrated that curcumin successfully improved parameters associated with oxidative stress (decreased the level of pro-oxidative biomarkers: malondialdehyde (MDA), 8-hydroxy-2-deoxyguanosine, enhanced concentrations of the antioxidants superoxide dismutase, catalase, and reduced glutathione) and inflammation and lowered uric acid to nearly normal levels [76]. Al-Rubaei et al. [76] implied that the urate-lowering effect of curcumin could be related to its potent antioxidant capacity.

#### 2.1.3. Human Studies

There are hardly any reports on the beneficial effects of curcumin in CKD patients. CKD plays a role in macrophage migration and enhanced endothelial trapping of macrophages [77]. Augmented macrophage infiltration into the kidney and other tissues contributes aggravated inflammation via greater release of pro-inflammatory cytokines. Numerous human studies have demonstrated that TGF-β mediates several crucial tubular pathological events, such as epithelial to mesenchymal transition, fibroblast proliferation, tubular and fibroblast extracellular matrix (ECM) production, and epithelial cell death leading, in the course of CKD progression, to tubular cell deletion and interstitial fibrosis [3,78]. A randomized, double-blind, and placebo-controlled study comprising patients with overt type 2 diabetic nephropathy revealed a significant decrease in plasma TGFβ following curcumin treatment [78]. Short-term turmeric supplementation was shown to be able to attenuate proteinuria, TGF-β, and IL-8 in these patients. Moreover, it turned out to be safe for these patients. Moreillon et al. [69] assessed the impact of herbal supplement composed of *Curcuma longa* and *Boswellia serrata* on systemic inflammation and antioxidant status in non-dialysis CKD patients. They observed a marked reduction of inflammatory markers TNFα, IL-6, and C-reactive protein, but no impact on creatinine and blood urea nitrogen (BUN). The antioxidant properties of curcumin have been confirmed in a double-blind study of diabetic and non-diabetic proteinuric CKD patients [60]. Jiménez-Osorio et al. [60] found that curcumin diminished lipid peroxidation in patients with non-diabetic proteinuric CKD (*p* < 0.05) and increased antioxidant capacity in those with diabetic proteinuric CKD (*p* < 0.05). However, it did not affect antioxidant enzymes activities nor Nrf2 activation. Moreover, they failed to show any impact of curcumin on proteinuria, estimated glomerular filtration rate, or lipid profile. Some studies have demonstrated that curcumin can block 5-lipoxygenase (5-LO), an enzyme which increases oxidative stress and is greatly activated in peripheral mononuclear blood cells of CKD patients and those on dialysis therapy [79,80]. Curcumin administration has also been suggested to be beneficial for renal transplant patients. In a randomized controlled trial, curcumin stimulated functional recovery of a kidney transplanted from a cadaver [61]. HD-dependent cadaveric kidney recipients who received a curcumin-based supplement for one month after surgery presented improved early graft function. Moreover, acute rejection and neurotoxicity were demonstrated to be the lowest in the high dose group. Authors suggested that the beneficial impact of bioflavonoids on early outcomes in cadaveric renal transplantation could be associated with the induction of heme oxygenase-1 (HO-1), which is an enzyme involved in an anti-inflammatory response and modulates apoptosis [61].

In CKD and HD patients, curcumin acts as a scavenger of free radicals, which results in a significant suppression of oxidative stress [81]. Due to the fact that curcumin has strong anti-inflammatory and pro-apoptotic properties, it has been tested for its potential utility in the treatment of osteoarthritis (OA) and rheumatoid arthritis (RA), which are diseases associated with degenerative changes in the joint, loss of its function, pain, and severe disability [82,83]. Excessive production and release of pro-inflammatory interleukin-1β (IL-1β), interleukin-6 (IL-6), and tumor necrosis factor-α (TNF-α) are observed in the course of OA [84]. Curcumin was shown to inhibit IL-1β-induced activation of NF-κB in human articular chondrocytes hampering caspase-3 activation and poly (ADP-ribose) polymerase PARP cleavage as well as COX-2 production, which all resulted in anti-apoptotic effects on IL-1β stimulated human chondrocytes [85,86]. Buhrmann et al. [83] revealed that curcumin suppressed IL-1β-induced catabolic signaling cascade in the mesenchymal stem cell (MSC) and chondrocytes. Moreover, curcumin has been suggested to block multiple sites within a TGF-β signaling cascade as well as down-regulate Smad in human proximal tubule cells [87,88].

Another study of the effect of curcumin in OA demonstrated that it considerably repressed matrix metalloproteinase (MMP)-13 mRNA and suppressed NF-κB pathway activation via the inhibition of I-κBα phosphorylation and degradation as well as P65 nuclear translocation likely diminishing inflammation [89,90,91]. Wang et al. [90] who examined the thesis that HA/chitosan nanoparticles (CNP) improved the efficiency of delivered curcuminoid observed that such composition stimulated the expression of IκB as well as promoted collagen II expression [90]. In vitro study of human peripheral blood mononuclear cells demonstrated that the combination of *L. acidophilus*, vitamin B, and curcumin efficiently downregulated Th17 cells and the related cytokine IL-17, which led to the enhanced expression of Treg-related cytokine IL-10 [82]. Moreover, in an animal model of OA, this combination reduced the expression of the catabolic matrix metalloproteinase-13 (MMP-13) and pro-inflammatory cytokines as well as upregulated anabolic tissue inhibitors of metalloproteinases (TIMPs), resulting in the decreased pain, the preservation of cartilage, the inhibition of osteoclastogenesis, and the regulation of anabolic/catabolic imbalance. Therefore, it seems that *L. acidophilus*, vitamin B, and curcumin have therapeutic potential in patients with OA [82]. However, we failed to find any study of therapeutic effect of curcumin in CKD patients with OA or RA.

#### 2.1.4. Resveratrol

Resveratrol (RSV), which is a stilbene derivative, is one of the most known and well analyzed polyphenols in various study populations [12]. This compound is abundant in grapes and red wine. Numerous studies have demonstrated its strong anti-oxidative, anti-hyperglycaemic, and anti-inflammatory properties [92]. The results of experimental in vitro as well as in vivo studies have indicated that resveratrol at doses contained in wine (light to moderate drinking) hampered inflammation, decreased oxidative stress, and restored NO production [93,94,95,96,97]. It also inhibited NOX-mediated production of ROS via the downregulation of gene expression and limited the activity of the oxidase. Resveratrol decreases mitochondrial superoxide production and, at the same time, it enhances the expression of numerous antioxidant enzymes via histone/protein deacetylase SIRT-1 or by nuclear factor E2-related factor 2 [98].

#### 2.1.5. In Vitro/Animal Studies

Beneficial impact of resveratrol on endothelial function was shown to be associated with the activation of deacetylase enzyme silent information regulator 2/sirtuin 1 (SIRT1) [99,100,101,102]. Moreover, this compound can also induce and activate the expression of eNOS, modulate oxidative stress, and inhibit inflammatory mediators [97,103,104,105]. Resveratrol enhances eNOS expression and activity via the simultaneous activation of SIRT1-AMPK and endoplasmic reticulum pathways as well as the stimulation of the l-arginine/NO/cGMP pathway, which results in higher NO bioavailability [106]. Other studies have indicated that RSV enhanced the expression of Krüppel-like factor 2 (KLF2) in human vascular endothelial cells leading to orchestrated regulation of transcriptional programs related to the endothelial vaso-protective phenotype [107]. The stimulation of KLF2 expression was associated with the activation of SIRT-1 and the synthesis of eNOS [97,105,108]. Mitogen-activated protein kinase 5/myocyte enhancing factor 2-dependent signaling pathway has been found to be involved in KLF2 upregulation by resveratrol [107]. In rats with streptozotocin (STZ)-induced diabetes, the administration of resveratrol considerably abated diabetes-related vascular dysfunction [108]. This effect was associated with the beneficial impact of resveratrol on oxidative stress markers as well as an RSV-related decrease in the aortic expression of TGF-β, increase in heme oxygenase-1 activity, NOS3 expression, and aortic nitrite concentration. Chen et al. [109] demonstrated that resveratrol attenuated trimethylamine-N-oxide (TMAO)-induced atherosclerosis by decreasing TMAO levels and enhanced hepatic bile acid neo-synthesis as the result of gut microbiota remodeling. It was found that enterohepatic farnesoid X receptor (FXR)-fibroblast growth factor 15 (FGF15) axis partially mediated in bile acid neo-synthesis. Furthermore, resveratrol enhanced the activity of bile salt hydrolase and subsequent formation of unconjugated bile acid as well as increased fecal bile acid loss, which resulted in the stimulation of hepatic CYP7A1 expression and higher hepatic bile acid synthesis. In consequence, diminished hepatocyte and plasma cholesterol levels as well as hampered atherosclerosis were observed [109]. In turn, Wellman et al. [110] revealed that resveratrol regulated gut microbiota (it increased the levels of anti-inflammatory Lactobacillus) by stimulating intestinal epithelial sirtuin-1 (SIRT1) and, therefore, it inhibited intestinal inflammation. The upregulation of Sirt1 expression also prevents the destruction of cartilage [111,112]. Sirt1 exerts favorable effects on cartilage by promoting chondrocyte survival, especially under stress conditions [113]. Resveratrol not only inhibited NF-κB activation simulated by TNF-β or T-lymphocytes in chondrocytes but also down-regulated NF-κB-dependent gene products involved in signaling pathways associated with cell proliferation, inflammation, degradation of the matrix, and apoptosis [114]. Buhrmann et al. [114] revealed that treatment with resveratrol protected mesenchymal stem cells against TNF-β-induced degradative and apoptotic morphological alterations. In turn, Limagne et al. [115] demonstrated that resveratrol-associated reduction in IL-6 secretion depended on NF-κB inhibition in chondrocytes.

#### 2.1.6. Human Studies

Resveratrol seems to be a promising nutraceutical, which could improve vascular functions in patients with chronic kidney disease [12]. A cross-sectional logistic regression analysis of National Health and Nutrition Examination Survey (NHANES) revealed that light wine consumption (<1/day) considerably decreased CKD prevalence and diminished the rate of CVD in patients with CKD after adjusting for age, race, sex, diabetes mellitus, hypertension, and cholesterol levels [116]. However, there are no randomized, placebo-controlled, large clinical trials comprising CKD patients, in which this hypothesis could be confirmed.

Some other studies indicated that, after the consumption, the bioavailability of polyphenols (resveratrol and curcumin) in the blood was considerably increased, especially in CKD patients in whom plasma levels of polyphenol metabolites remained elevated for a long time as a result of renal impairment and the inability to adequately excrete these metabolites [117]. Such sustained bioavailability of polyphenols in the blood may enable their prolonged action on the vasculature. However, more research is needed to warrant that it is safe and no distant adverse effect will occur.

Resveratrol have also been suggested to be effective in OA. An in vitro study confirmed anti-inflammatory and chondroprotective effects of resveratrol in human chondrocytes [114,118].

#### 2.1.7. Green Tea and Coffee

Green tea is a source of catechins—polyphenolic compounds (flavonoids) showing antioxidant, anti-inflammatory, and anti-carcinogenic properties [15,119,120,121]. Due to the fact that the presence of inflammation and oxidative stress contribute to the development and progression of renal diseases, it has been suggested that frequent consumption of green tea or green tea extracts could have a favorable effect on renal function.

#### 2.1.8. In Vitro/Animal Studies

The mechanism of the inhibition of pro-inflammatory and pro-apoptotic oxidative injury by catechins involves the decrease in the reactive oxidative species (ROS) production, the translocation of NF-kB and activated protein 1, and the expression of intercellular adhesion molecule 1 (ICAM-1) [122].

Wang et al. [123] examined reno-protective properties of active polyphenol-epigallocatechin-3-gallate (EGCG) contained in green tea in a unilateral ureteral obstruction (UUO) mice model. After 14 days of EGCG administration (50 mg/kg/day), significantly improved renal function and the restoration of UUO-induced kidney weight loss were observed. Moreover, this active compound limited UUO-induced oxidative stress and inflammatory responses, induced by both NF-κB and Nrf2 nuclear translocation in the UUO kidney as well as stimulated heme oxygenase-1 (HO-1) production. Authors suggested that NF-κB and Nrf2 signaling pathway regulation might be involved in a reno-protective effect of EGCG [123]. In turn, Wongmekiat et al. [124] demonstrated that treatment with catechins considerably weakened disturbances caused by cadmium. In their study, cadmium-intoxicated rats had significantly increased blood urea nitrogen and creatinine, decreased creatinine clearance, reduced levels of antioxidant thiols, superoxide dismutase, and catalase, and developed renal pathologies including severe tubular damage, apoptosis, and abnormal mitochondrial structure. Catechins administration alleviated these effects and it seems plausible that they effectively protect the kidney against the toxic effect of cadmium due to its antioxidant, anti-inflammation, and mitochondrial protection properties [124].

#### 2.1.9. Human Studies

Additionally, in humans, catechins administration brings favorable effects. Daily supplementation with 455 mg of catechins extracted from decaffeinated green tea (equivalent of four cups of green tea/day) was demonstrated to lower ROS production, diminish hemodialysis-enhanced plasma hypochlorous acid activity and pre-dialysis plasma hydrogen peroxide activity, and lower phosphatidylcholine hydroperoxide, C-reactive protein, and pro-inflammatory cytokine concentrations [125]. Due to the fact that oxidized pro-atherosclerotic products, including oxidized LDL and phosphatidylcholine hydroperoxide, cannot be mechanically removed during hemodialysis, the use of potent antioxidants limiting ROS generation and protecting against oxidative damage seems to be beneficial in this group of patients, as it could slow down the progression of atherosclerotic vascular disease [15]. The consumption of green tea (5 g/day) by patients with CKD was associated with significantly improved FMD in the catechin group. However, this study failed to find considerable improvement in clinical characteristics, oxidative stress, inflammatory markers, and circulating endothelial progenitor cells (EPCs) number in CKD requiring dialysis [126]. Recent systemic review and meta-analysis suggested that green tea could be beneficial in the management of obesity [127]. It demonstrated that the administration of green tea significantly reduced body weight (WMD: −1.78 kg, 95% CI: −2.80, −0.75, *p* = 0.001) and body mass index (BMI) (WMD: −0.65 kg/m^2^, 95% CI: −1.04, −0.25, *p* = 0.001). Waist circumference was considerably lower in participants consuming ≥800 mg/day of green tea for <12 weeks. Most significant body weight reduction was observed when green tea intake was <500 mg/day for 12 weeks [127].

#### 2.1.10. Caffeine

Caffeine is another active compound, which have been suggested to act favorably on kidneys. However, the available information is sparse [15].

#### 2.1.11. Human Studies

In the study conducted by Jhee et al. [128], the consumption of one cup per day (HR, 0.76, 95% confidence interval, 0.63–0.92) and ≥2 cups per day (HR, 0.80, 95% confidence interval, 0.65–0.98) was shown to reduce the risk of chronic kidney disease development in healthy consumers compared with non-drinkers. In addition, in healthy participants of the Doetinchem Cohort Study, the consumption of coffee was associated with a slightly higher eGFR, but this effect was not associated with glomerular hyperfiltration [129].

#### 2.1.12. Animal Studies

However, some experimental data suggest that caffeine may exert an adverse impact on renal function in patients with pre-existing renal dysfunction and high-renin hypertension with an increase in proteinuria [130]. According to Tofovic et al. [130], the negative impact of caffeine on renal function in the presence of hypertension could be ascribed to its capacity to block renal adenosine receptors, resulting in the augmentation of angiotensin II-induced glomerular hypertension.

### 2.2. Vitamin D Supplementation

Vitamin D can come from diet (ergocalciferol {D2} or cholecalciferol {D3} forms), cutaneous synthesis involving the absorption of UVB solar radiation (290–315 nm), during which 7-dehydrocholesterol converted to pre-vitamin D3 and, in the next steps, to cholecalciferol by thermal isomerization or from supplements [131,132]. It is common knowledge that the prevalence of vitamin D deficiency is very high in CKD, as it worsens with the progressive loss of renal function, reaching more than 80% in pre-dialysis patients [133,134]. The exact mechanisms of this phenomenon are not fully understood.

The deficiency of vitamin D is associated with secondary hyperparathyroidism (SHPT), diabetes, high blood pressure, endothelial function, neoplastic diseases, and autoimmune diseases as well as the regulation of the cell cycle [132,134]. Studies have also demonstrated a strong inverse correlation between the serum vitamin D level, morbidity, and mortality in this population [131,135,136]. Due to the fact that vitamin D plays a crucial role in the regulation of mineral and bone metabolism as well as exerts an impact on cardiovascular and immune systems, it is believed that its supplementation is important for human health. Kidney Disease: Improving Global Outcomes (KDIGO) guidelines from 2017 recommend the correction of vitamin D deficiency and insufficiency in CKD with GFR <60 mL/min/1.73 m^2^ using strategies provided to the general population [137]. In more advanced stages, such as in pre-dialysis patients, native forms of vitamin D should be used, while those with more severe or progressive phases of SHPT ought to be administered calcitriol and its analogs. However, these guidelines do not set any reference value for the 25(OH)D level, but recommend its evaluation in patients at stages of CKD above 3 with progressively increasing or persistently elevated (above upper normal) PTH levels. It should be kept in mind that extra high levels of native forms of vitamin D can show toxicity, including hypercalcemia and hyper-phosphataemia.

#### Human Studies

A double-blind randomized clinical trial (RCT) assessing the impact of oral supplementation with calcifediol on SHPT treatment in pre-dialysis CKD patients demonstrated that this method is safe and effective [138]. Another multicenter study of patients with stage 3 or 4 CKD, SHPT, and vitamin D insufficiency showed that the PTH lowering effect of ER calcifediol was independent of the CKD stage and such treatment did not bring any adverse events [139].

Numerous studies have also indicated that vitamin D deficiency is related to cardiovascular disease, subclinical peripheral arterial disease (independently of other traditional or non-traditional atherosclerosis risk factors), and atherosclerosis [140,141]. According to meta-analysis performed by Pilz et al. [140], every 10 ng/mL of decrease in the vitamin D level is associated with an increase of 14% in risk of all-cause mortality in patients with CKD. Vitamin D administration was also demonstrated to diminish cardiovascular mortality risk in 27% of CKD patients [142]. Moreover, observational cohort Health, Aging, and Body Composition (Health ABC) Study found an independent relationship between lower calcitriol levels and kidney function decline in community-living, older adults [143]. The results of a randomized, double-blinded, placebo-controlled clinical trial in which patients with nondiabetic CKD stage 3–4 and serum 25(OH)D level ≤20 ng/mL were administrated either cholecalciferol (300,000 IU) or a matching placebo indicating that oral cholecalciferol considerably enhanced endothelium-dependent brachial artery flow-mediated dilation, thus, ameliorating vascular function and the changes correlated with 25(OH)D levels [144]. Moreover, it has been suggested that a protective effect of vitamin D on the arterial wall involves the inhibition of smooth muscle cell proliferation and the reduction of pro-inflammatory cytokines (tumor necrosis factor, interleukin-6) resulting in the lowering of vascular inflammation [134,141]. The study assessing the impact of oral cholecalciferol on cardiac parameters and biomarkers for endothelial cell activation in children with CKD revealed significantly ameliorated flow-mediated dilatation (FMD) and local arterial stiffness after vitamin D supplementation [145]. However, a randomized controlled trial Paricalcitol Capsule Benefits in Renal Failure-Induced Cardiac Morbidity (PRIMO) comparing paricalcitol with placebo failed to observe any impact of active vitamin D on a left ventricular mass index and diastolic function in CKD patients [146]. In addition, in the Effect of Paricalcitol on Left Ventricular Mass and Function in CKD—The OPERA Trial, the administration of paricalcitol had no effect on LV mass index and LV mass regression in patients with CKD stages G3a–G5 and left ventricular (LV) hypertrophy [147].

The inconsistencies in the results of studies are associated with the differences in baseline levels of serum 25(OH)D in the choice of a vitamin D form and dose, the time of treatment, and adherence to supplementation as well as the presence of polymorphisms within the vitamin D receptor (VDR) gene [148,149].

### 2.3. Polyunsaturated Fatty Acids (PUFA)

Polyunsaturated fatty acids (PUFAs) include ω-3 PUFAs derived from plants or meat and ω-6 PUFAs derived from plants or marine creatures [150]. PUFAs have been demonstrated to possess anti-inflammatory properties, which can, among others, protect kidneys from damage [151,152]. Dietary intake of polyunsaturated fatty acids may also be advantageous in the prevention of the progression of CKD [16].

#### 2.3.1. Animal Studies

The results of studies performed on rodent models of diabetic nephropathy and hypertension indicated that the administration of polyunsaturated or monounsaturated fatty acids in the diet reduced glomerulosclerosis and glomeruli loss [153,154]. Study of 5/6 nephrectomized male Wistar rats fed an n-3 PUFA-enriched diet for six weeks revealed improved endothelial-dependent vasodilation (*p* < 0.05) compared with CKD rats on a standard diet [155]. The blockade of eNOS by L-NAME was found to worsen vasodilation. Moreover, increased expression of eNOS (*p* < 0.05) and reduced expression of NOX4 (*p* < 0.05) as well as diminished concentration of 3-nitrotyrosine levels were observed in this study. These findings demonstrated that n-3 PUFA ameliorate endothelial dysfunction by restoring NO bioavailability in CKD [155].

#### 2.3.2. Human Studies

Numerous studies have confirmed the importance of polyunsaturated fatty acids in the diet since they cannot be synthesized by the human body, and they exert favorable effects in CKD patients [16,156,157]. The results of secondary analysis of the Diabetes Control and Complications Trial revealed that higher dietary eicosapentaenoic acid and docosahexaenoic acid consumption was inversely associated with the degree but not with the incidence of albuminuria in type 1 diabetes [158]. However, larger studies are required to confirm the results due to suboptimal quality of the studies included in the meta-analysis. Such findings are promising as even a minimal drop in urinary albumin excretion has been demonstrated to be associated with decreased risk of kidney failure and cardiovascular disease [159]. The study of impact of omega-3 fatty acid containing 160 mg eicosapentaenoic acid (EPA) and 100 mg docosahexaenoic acid (DHA) demonstrated mixed results with respect to cardiovascular disease risk. The addition of fish oil to the diet of hemodialysis patients was associated with a considerable increase in HDL levels, but, at the same time, with the elevation of LDL concentrations compared with corn oil (source of omega-6 fatty-acid) [160]. In turn, a larger, randomized clinical trial of the effect of omega-3 PUFA supplementation (45% EPA and 37.5% DHA) in hemodialysis patients indicated a significant decrease in serum triglycerides in the n-3 PUFA group compared with olive oil (control supplement) (*p* = 0.01) [161]. However, no significant impact was seen on total cholesterol, high-density lipoprotein (HDL) cholesterol, low-density lipoprotein (LDL) cholesterol, Lp(a), or apoB. In the other study, the supplementation of omega-3 (2 g/day) compared with a similar dose of olive oil had no effect on lipids concentration, but it diminished systolic, diastolic, and mean blood pressure [162]. The results of studies concerning polyunsaturated fatty acids are not consistent. However, it seems that the intake of omega-3 fatty acids may be effective in lowering the risk of cardiovascular disease in HD patients via its impact on risk factors, such as blood pressure, LDL, HDL, and triglycerides levels [16].

### 2.4. Conjugated Linolenic Acid (CLA)

Conjugated linolenic acid (CLA) is synthesized naturally in grass-fed ruminants (cattle, sheep, goats) by fermentative bacteria *Butyrivibrio fibrisolvens* [16,163]. CLA is a mixture of isomers of linoleic acid with conjugated double bonds. Conjugated linolenic acid is present in plant seeds, including pomegranate seed, catalpa seed, Tung seed, bitter gourd seed, trichosanthes seed, pot marigold seed, snake gourd seed, and jacaranda seed [16]. In humans and animals, CLA can be metabolized into CLA [164].

#### Animal Studies

The results of animal studies have indicated that CLA possess anti-inflammatory, anti-atherosclerotic, and anti-diabetic properties [16,165]. Moreover, it was shown to reduce renal production of PGE2, and to have significant renal anti-inflammatory and anti-fibrotic effects. Therefore, it seems that CLA may be a useful compound in dietary amelioration of renal disease [16,165].

### 2.5. Red Yeast Rice and Berberine

Red yeast rice and berberine are considered to be lipid-lowering nutraceuticals [166]. Both these compounds have been demonstrated to improve endothelial function. However, their effect on pulse wave velocity (PWV) has not been widely analyzed, especially in CKD patients. Red yeast rice decreases cholesterolemia directly via the inhibition of liver 3-hydroxy-3-methyl-glutaryl-coenzyme A reductase, while berberine hinder the cleavage of the receptor for LDL-cholesterol from the liver cell membranes [167].

#### Human Studies

Cicero et al. [166] demonstrated that both analyzed nutraceutical used in combination effectively diminished LDL-C and TG by 24% and 21%, respectively. They also confirmed the efficacy as well as safety of this “treatment” in patients with mild-to-moderate CKD. Moreover, they revealed that this approach improved PWV in both non-CKD and CKD patients. In addition, Affuso et al. [168] reported that the combination of low-dosed red yeast rice and berberine resulted in direct protective vascular effects, including the improvement of endothelial dysfunction and also insulin sensitivity. Moreover, berberine has also been found to be effective in a body weight decrease. Recent meta-analysis found that berberine intake resulted in a significant reduction of body weight, BMI, waist circumference, and CRP levels in patients with metabolic diseases [169]. Such treatment considerably diminished body weight (WMD = −2.07 kg, 95% CI −3.09, −1.05, *p* < 0.001), body mass index (BMI) (WMD = −0.47 kg/m2, 95% CI −0.70, −0.23, *p* < 0.001), waist circumference (WC) (WMD = −1.08 cm, 95% CI −1.97, −0.19, *p* = 0.018), and C-reactive protein (CRP) concentrations (WMD = −0.42 mg/L, 95% CI −0.82, −0.03, *p* = 0.034).

### 2.6. Menaquinone-7 (MK-7)

Western diets are somewhat rarely the source of vitamin K2 [170]. This vitamin is present in fermented foods, egg yolk, chicken, beef liver, and natto (Japanese food fermented with the use of some bacteria).

#### 2.6.1. In Vitro/Animal Studies

Yasufumi et al. [171] in their in vitro study (of human hepatocarcinoma cell line) observed that MK-7, at a concentration above 7.5 µM, was a strong inhibitor of cholesterol biosynthesis. Lupo et al. [172] found that the combination of nutraceuticals significantly affected cholesterol metabolism and its administration could enable the control of mild hypercholesterolemia in CKD patients. In their in vivo study of uremic rats, the supplementation with a formulation containing MK-7 (3.5 µg/g of diet), MgCO_3_ (3.7 µg/g of diet), and Sucrosomial^®^ Iron (1 mg/g of diet) possessed hypo-cholesterolemic action and it turned out that it was MK-7, which was responsible for cholesterol-inhibition activity. This strong hypo-cholesterolemic activity could be partly ascribed to MK-7-related hampering of PCSK9 secretion. Berberine exert a similar effect on cholesterol-related genes. However, due to the fact that these two compounds entirely differs in their chemical structures, it is rather unlikely that their actions are mediated via the same pathways. Based on the fact that the MK-7 particle very closely resembles the chemical structure of squalene (cholesterol precursor in the mevalonate pathway), Lupo et al. [172] suggested that MK-7 may stimulate an inhibitory action on the catalytic activity of squalene synthase catalysing the first step in cholesterol biosynthesis, thus, playing, under certain conditions, a regulatory function.

#### 2.6.2. Human Studies

Inadequate levels of vitamin K2 result in higher probability that calcium will be deposited in vessels (vascular calcification) instead of the bone matrix, which increase the risk of cardiovascular disease and worsen the bone state. The results of population-based Rotterdam study indicated that low levels of vitamin K2 was associated with increased prevalence of severe aortic calcification and higher mortality [173]. Vitamin K deficiency was found to be an important risk factor of overall mortality in kidney-transplanted patients [174]. Therefore, the question appears regarding whether people, especially CKD patients (in whom bone disorders are frequent), could benefit from the supplementation with vitamin K. Kurnatowska et al. [175] observed that the administration of MK-7 (90 μg) together with 10 μg of cholecalciferol in patients with CKD stages 3-5 altered levels of calcification promoters and inhibitors, including desphosphorylated-uncarboxylated MGP (dp-ucMGP), osteoprotegerin (OPG), and osteocalcin (OC), diminishing the progression of atherosclerosis, which implies that vitamin K can correct the biochemical and local tissue consequences of vitamin K deficiency [176]. In addition, in patients undergoing dialysis, the supplementation with vitamin K seems to be of high importance. The results of randomized controlled trial demonstrated that MK-7 administration reduced dp-uc-MGP and protein induced by vitamin K absence II (PIVKA-II) levels, decreasing vascular calcification [177].

Table 1 summarizes the results of animal and human studies concerning the effects of nutraceuticals.

## 3. Combined Use of Nutraceuticals

The use of high doses of nutraceutical may raise some tolerability concerns regardless of whether they will or will not be associated with side effects similar to that observed in the case of statins or metformin [178]. The combination of nutraceuticals with a different but synergic mechanism of action at lower and safer dosages seems to be a good solution. This thesis was confirmed by the result of some studies indicating nutraceuticals superior performance when co-delivered, which could be associated with synergism observed in the case of various bioactive substances [179]. Phytotherapy, which is based on combined activities of a mixture of constituents, opens new treatment opportunities [180]. Numerous studies have demonstrated that nutraceuticals participate in macro-mechanistic processes, including inflammation, oxidative stress, and sympathetic over-activation, and upregulate NO bioavailability and antioxidant protection [12,15]. They enhance endothelial function, decrease arterial stiffness, inhibit platelet aggregation, lower blood pressure, limit the release of the potent vasoconstricting peptide endothelin-1, modulate transcriptional networks, and/or signaling cascades. Some nutraceuticals target one specific pathway, while other (resveratrol and curcumin) exhibit a multitude of biologically active properties [12]. The use of active compound mixtures not only enable multitargeting treatment, but also the pharmacological effects exerted by these components may involve synergistic or antagonistic interactions [180]. Herbal mixtures have been shown to protect active substances from decomposition by enzymes, activate pro-drugs, or deactivate active compounds to inactive metabolites, interfere with cellular transport (modifications of transport across membranes of cells or organelles), act synergically at different points of the same signaling cascade (multi-target effects), inhibit the binding to target proteins, and evade multi-drug resistance mechanisms [180,181]. The nature of many diseases is multi-factorial and is mediated by various cellular pathways. Therefore, it seems that preparations with a wide range of activities could prove effective in their treatment [181]. Drug-related inhibition of a specific target sometimes results in the switching of alternate routes, which is associated with the development of resistant cells or resistant organisms and subsequent drug resistance and clinical failure of the drug. Multicomponent therapy allows using beneficial actions of many compounds at the same time while increasing the change of success (recovery). The results of some studies confirm the effectiveness of an active ingredients mixture. Combination of curcumin and quercetin was demonstrated to reduce the number and size from the baseline of polyps in patients with familial adenomatous polyposis [182]. In turn, Oxy-Q bioflavonoid therapy with curcumin and quercetin ameliorated early graft function, lowered the risk of acute rejection and neurotoxicity (high dose group), and improved early outcomes in cadaveric renal transplantation, likely via HO-1 induction [61]. In addition, the combination of natural compounds with some therapeutic drugs may prove beneficial. A prospective randomized study of the effects of quercitin and curcumin (FlogMEV) in combination with prulifloxacin on chronic bacterial prostitis revealed that nutraceuticals enhanced clinical efficacy of the drug [183]. However, some nutraceuticals can also be toxic at high doses and their activity may result in the accumulation of some drugs, strengthening drug’s effects, and consequent occurrence of side effects (sometimes severe or life-threatening).

## 4. Are Nutraceuticals Always Safe?

Healing properties of many plants (ginseng, cinnamon, thyme, garlic, cumin, turmeric, etc.) have been known for ages [184] and this have triggered a series of studies of nutraceuticals. The global market of nutraceutical products has dramatically risen recently due to their great popularity and due to the fact that there are no regulations to control them [185]. Nutraceuticals are frequently advertised as being effective and completely safe (devoid of any side effects). However, this is not always the truth. Nutraceuticals may have poor solubility, low permeability, and fast metabolism and there are not enough clinical trials assessing their safety and efficacy [184,186]. Moreover, herbal products, particularly complex mixtures derived from herbal plants, contain bioactive fractions or compounds that are difficult to characterize and to normalize the content due to natural variation in the plant composition, which is associated with issues in controlling the intake of such preparations [187]. The shortage of clinical trials, which is associated with problems with the implementation of a restricted dietary intervention, translates into a lack of dosage recommendations and general regulations that could enable the control of nutraceuticals.

In case of some nutraceuticals, the occurrence of side effects and toxicity have been described. First of all, nutraceuticals can be contaminated with pesticides, fertilizers, metals, or other toxic plants [188]. Second, the intake of high doses of nutraceuticals for a long time can be associated with side effects. For example, extra high consumption of resveratrol was shown to result in mild diarrhea, nausea, hypersensitivity, and anal pruritus [189]. Some herbs, e.g., juniper berries and bucha leaves, which have a potential as diuretics, may lead to “kidney irritation” or damage [190].

**Figure 1:**
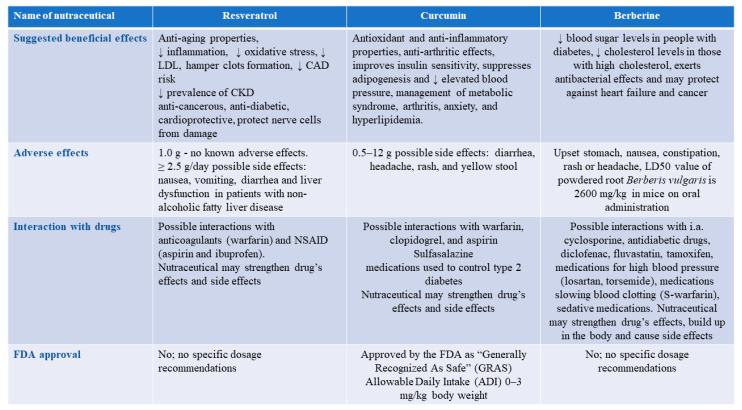
The summary of Resveratrol, curcumin, and berberine effects and interactions.

Moreover, herbal products contain many phytochemicals, which can be associated with unpredictable drug-herbal interaction after co-administration [191]. These interactions may result in either inhibition of the drug efficacy (and, therefore, its low effectiveness) or strengthened drug’s effects (leading to higher incidence of toxicity and severe side effects). For example, ginkgo biloba, which is indicated for schizophrenia, Alzheimer’s disease, dementia, and cerebral insufficiency, could also enhance the risk of spontaneous bleeding or even carcinogenicity if administered at high doses for a long time [192,193]. In addition, green tea, which is believed to prevent obesity and metabolic disorders and even to have anti-cancer properties, has been shown to be nephrotoxic, hepatotoxic, and reproductive toxic if over-administrated [194,195]. Many commonly consumed plant products (e.g., aloe vera, cinnamon, caffeine can exert deleterious effects, such as genotoxicity, hepatotoxicity, and mutagenicity). Transplant patients are a high-risk group due to the fact that adverse interactions between herbs and medications could potentially increase the risk for rejection or loss of the kidney. According to NFK, the following herbs may be toxic to the kidneys: Artemisia absinthium (wormwood plant), Autumn crocus, Chuifong tuokuwan (Black Pearl), Tung shueh, and Horse chestnut. In turn, Ginger, Aloe, Ginseng, Horsetail, Blue Cohosh, Broom Dandelion, Coltsfoot, Mate, Licorice, Senna, and other may be harmful in chronic kidney disease [190]. Moreover, as we mentioned above, nutraceutical-drug interactions may be associated with serious, sometimes life-threatening, events when the first one interferes with a drug’s metabolic pathway or affects its transporters [196]. Due to a lack of appropriate pharmacodynamics as well as pharmacokinetic and safety studies for nutraceuticals, it is a challenge to foresee the occurrence of such interactions [184]. Herbal products comprising turmeric, chamomile, ginger, garlic, and ginkgo have been demonstrated to exert an impact on the actions of aspirin and some non-steroidal anti-inflammatory drugs enhancing the risk of bleeding associated with the inhibition of platelet aggregation ability [197]. KDIGO guidelines reflect the awareness of the unpredictability of nutraceuticals side effects and possible toxicity. Therefore, according to the summary of the 2020 KDIGO Diabetes Management in CKD Guideline: evidence-based advances in monitoring and treatment [198] physicians should “review the patient’s history of the use of over-the-counter nonsteroidal anti-inflammatory drugs, supplements, and herbal treatments, and patients should be counseled to discontinue these remedies if present.” Moreover, due to the fact that some supplements can exert unwanted effects before, during, and after surgery, the healthcare provider should be informed about them. Additionally, the earlier summary of KDIGO 2012 CKD Guideline: behind the scenes, need for guidance, and a framework for moving forward recommend “that adults with CKD seek medical or pharmacist advice before using over-the-counter medicines or nutritional protein supplements. (1B)” and suggest “not using herbal remedies in people with CKD. (1B)” [199]. Furthermore, they even suggest “not to routinely prescribe vitamin D supplements or vitamin D analogs, in the absence of suspected or documented deficiency, to suppress elevated parathyroid hormone concentrations in people with CKD not on dialysis. (2B)”. Figure 1 presents summary of given nutraceutical effects and interactions.

## 5. Conclusions

The results of in vitro and animal studies of kidney injury have indicated that the use of some nutraceuticals can prove beneficial in the hampering of renal injury progression and in the lowering of cardiovascular risk. These bioactive compounds could reduce the inflammation, oxidative stress, and sympathetic overactivation, promote renal blood flow and glomerular filtration rate, upregulate antioxidant properties, and rise NO bioavailability as well as inhibit interstitial fibrosis, and stimulate tubular regeneration. However, the evidence from human studies is too sparse to draw any final conclusions. Herbal products can be dangerous for CKD patients due to the fact that impaired kidneys are not able to clear waste products with similar efficacy as a healthy person [190]. Such patients should be aware of the fact that only a few nutraceuticals have been studied in CKD in few clinical trials enrolling small groups of participants. Encouraging results of animal studies cannot be translated directly into humans due to the fact that there are many differences between these species, studied animals do not have comorbidities, and they do not take human drugs. Our diet and lifestyle are completely different. Therefore, large clinical trials are required to find the answer to the question whether nutraceuticals (and which products exactly) can be used by patients with chronic kidney disease in order to slow down the development of kidney impairment-related disorders. Moreover, due to the lack of safety analyses, recommended doses are frequently not known. It is also difficult to assess the exact content of an active substance of interest in a natural product due to its variability. Natural products containing a given nutraceutical may also comprise other active substances, which are not recommended for CKD patients. Due to insufficient regulation of herbal supplements, there is no necessity to perform appropriate tests. Therefore, the purity, safety, and effectiveness of the products are unknown. That is why herbal preparations may be contaminated with toxic heavy metals, fertilizers, or pesticides. Bearing in mind all the above, patients with CKD (but not only they) should be cautious with unstandardized herbal extracts and consult a physician about dosage, safety, duration of use, and interactions with prescription drugs. Recommended dosage/time of use should never be prolonged. Patients should also avoid combining supplements with other supplementary, prescribed, or over-the-counter drugs and must not substitute natural products for prescribed medicines.

## Figures and Tables

**Table 1:** The summary of the effect of nutraceuticals use in CKD—the results of animal and human studies.

Type of Study/Study Group	Nutraceutical/Dose	Effects of Nutraceutical Administration	Ref
**Animal Studies**			
**Curcumin**			
Nephrectomized rats (5/6Nx)	Curcumin (120 mg/kg/day) dissolved in 0.05% CMC via oral gavages during 30 days	➢ Restored SBP, reduced interventricular and rear wall thickening, decreased LVSd, and restored EF. ➢ Diminished MMP-2 levels and overall gelatinase activity, reduced oxidative stress, and inhibited the mitochondrial permeability transition pore opening.	[59]
5/6 nephrectomized (Nx) rats	Curcumin (75 mg/kg) and enalapril (10 mg/kg), 10 weeks	➢ Reduced renal dysfunction. ➢ Decreased NF-α and IL-1β as well as PLA(2), COX 1, and COX 2. ➢ Curcumin ameliorates CKD by blocking inflammatory signals even if it is given at a later stage of the disease.	[66]
Sprague-Dawley rats with 5/6 nephrectomy (Nx) induced CRF/in vitro (mesangial cells)	Untreated (Nx), curcumin-treated (curcumin), and enalapril-treated (enalapril) groups	➢ The restoration of decreased anti-inflammatory PPARgamma in Nx animals. ➢ Dose-dependent antagonism of TNF-alpha-mediated decrease in PPARgamma and blockage of the transactivation of NF-kappaB and repression of PPARgamma. ➢ Anti-inflammatory property of curcumin may be responsible for alleviating CRF in Nx animals.	[71]
5/6 nephrectomized (Nx) Sprague-Dawley rats	(1) control (sham), (2) Nx, (3) Nx + curcumin (150 mg/kg bid), and (4) Nx + enalapril (15 mg/kg bid) as positive control, 7 weeks	➢ Significantly reduced proteinuria by 40–60%. ➢ Blunted the action of several key signaling molecules implicated in the hypertrophic response. ➢ Attenuated cardiac hypertrophy and remodeling (independent of SBP reduction) as a result of the deactivation of multiple hypertrophic signaling pathways.	[65]
Apoferritin-induced CSS model in Cfh-deficient (Cfh(-/-)) mice	Curcumin treatment (30 mg/kg) given every day	➢ Significant reduction in the number of splenic CD19(+) B cells and the ratio of CD19: CD3 cells (*p* < 0.05). ➢ Protective function against the apoferritin-related reduction in the M2 subset of splenic macrophages. ➢ Reduced mRNA expression of inflammatory proteins MCP-1 and TGF-β and matrix proteins, fibronectin, laminin, and collagen. ➢ Reduced glomerulosclerosis, improved kidney function.	[68]
**Resveratrol**			
Streptozotocin-diabetic rats	RSV (10 mg/kg) in presence or absence of an HO-1 blocker, Zinc protoporphyrin (ZnPP).	➢ Significantly abrogated diabetes-induced vascular dysfunction. ➢ This improvement was associated with the ability of RSV to decrease oxidative stress markers, a reduction in the aortic TGF-β expression, increased NOS3 expression, and aortic nitrite concentration and HO activity. ➢ These ameliorative effects were when ZnPP administered prior to RSV diminished these beneficial effects.	[108]
Female C57BL/6J mice and ApoE−/− mice with a C57BL/6 genetic background	Resveratrol	➢ Attenuated trimethylamine-N-oxide (TMAO)-induced atherosclerosis. ➢ Decreased TMAO levels via the inhibition of commensal microbial trimethylamine (TMA) production via gut microbiota remodeling. ➢ Increased levels of the genera Lactobacillus and Bifidobacterium.	[109]
**Green Tea and Coffee**			
Anesthetized rat bladder	Green tea extract (catechins), 2 weeks	➢ Reduced SP-induced bladder intercellular adhesion molecule expression and ROS amount and ameliorated hyperactive bladder response. ➢ Catechins pretreatment can ameliorate SP-induced neurogenic inflammation via the action of antioxidant, anti-adhesion, and anti-inflammatory activity.	[122]
Unilateral ureteral obstruction (UUO) mice model	Epigallocatechin-3-gallate (EGCG) (50 mg/kg/day), 2 weeks	➢ Restoration of UUO-induced kidney weight loss and renal dysfunction. ➢ Prevention of UUO-induced oxidative stress and inflammatory responses in the obstructed kidney. ➢ Induction of NF-κB and Nrf2 nuclear translocation in the UUO kidney and stimulation of heme oxygenase-1 (HO-1) production. ➢ Reno-protective effect of EGCG are mediated by NF-κB and Nrf2 signalling pathway regulations.	[123]
Male Wistar rats	Cadmium (CdCl2 2 mg/kg, i.p.) and cadmium plus catechin (25, 50, and 100 mg/kg, orally), 4 weeks	➢ Attenuated all the changes caused by cadmium. ➢ Effective protection of the kidney against toxic effect of cadmium, presumably via its antioxidant, anti-inflammation, and mitochondrial protection.	[124]
**Polyunsaturated fatty acids (PUFA)**			
5/6 nephrectomized male Wistar rats (CKD) and sham operated animals (SHAM)	n-3 PUFA enriched diet (CKD + PUFA, *n* = 10) vs. standard diet, 6 weeks.	➢ Improved EDD (*p* < 0.05). ➢ Blockade of eNOS by L-NAME worsened EDD. ➢ Increased (*p* < 0.05) eNOS and reduced (*p* < 0.05) expression of NOX4 and 3-nitrotyrosine levels. n-3 PUFA improve endothelial dysfunction by restoring NO bioavailability in CKD.	[155]
**Conjugated Linolenic Acid (CLA)**			
Han:SPRD-cy rat model of polycystic kidney disease (PKD), heterozygotes	Diets containing corn oil with a CLA enriched oil (1% of diet by weight as CLA) vs. corn oil (control), 8 weeks	➢ Reduced interstitial inflammation (*p* < 0.001), fibrosis (*p* = 0.03), and renal PGE2 release (*p* = 0.02). ➢ Increased renal and hepatic CLA isomers. ➢ Reduced hepatic linoleic acid proportion. ➢ No impact on renal proportion of the PGE2 precursor, arachidonic acid. ➢ Significantly increased hepatic AA proportion (*p* = 0.009).	[165]
**Human Trials**			
**Curcumin**			
Randomized double-blind placebo-controlled clinical trial of 101 Mexican patients with nondiabetic or diabetic proteinuric CKD	Placebo or 320 mg/day curcumin for 8 weeks	➢ No improvement of proteinuria, eGFR, or lipid profile. ➢ Attenuation of lipid peroxidation in individuals with nondiabetic proteinuric CKD (*p* < 0.05) and enhanced antioxidant capacity in subjects with diabetic proteinuric CKD (*p* < 0.05). ➢ No impact on the antioxidant enzymes activities or Nrf2 activation. Dietary supplementation with curcumin can reduce oxidative stress in patients with nondiabetic or diabetic proteinuric CKD.	[60]
43 dialysis dependent cadaveric kidney recipients	480 mg of curcumin and 20 mg of quercetin. Control (placebo), low dose (one capsule, one placebo), and high dose (two capsules).	➢ Bioflavonoid therapy improved early graft function. ➢ Acute rejection and neurotoxicity were lowest in the high dose group (control, 14.3% vs. low-dose: 14.3% vs. high-dose: 0%). ➢ Urinary HO-1 was higher in bioflavonoid groups. ➢ The improvement of early outcomes in cadaveric renal transplantation could be mediated by HO-1 induction.	[61]
16 patients with CKD	Herbal supplement composed of Curcuma longa and Boswellia serrata, or placebo.	➢ Significant time effect (*p* = 0.03) and time x compliance interaction effect (*p* = 0.04) for IL-6. ➢ No significant differences were observed for TNF-α and GPx. ➢ Curcumin and Boswellia serrata are safe and tolerable and improve the levels of an inflammatory cytokine.	[69]
A randomized, double-blind, and placebo-controlled study of 40 patients with overt type 2 diabetic nephropathy	Study group: 3 capsules (daily) with 500 mg turmeric (22.1 mg of active curcumin) for 2 months.Control group: placebo	➢ Significantly decreased serum levels of TGF-β and IL-8 and urinary protein excretion and IL-8 (pre-turmeric vs. post-turmeric supplementation values). ➢ No adverse effects related to turmeric supplementation were observed during the trial. ➢ Short-term turmeric supplementation can attenuate proteinuria.	[78]
**Resveratrol**			
Cross-sectional logistic regression of National Health and Nutrition Examination Survey (NHANES)	Wine. No consumption (0 glass per day), light (<1 glass per day), moderate (≥1 glasses per day).	➢ After adjusting for demographics and CVD risk factors, light wine consumption was associated with lower prevalence of CKD defined as UACR ≥30 mg/g. ➢ Light wine consumption was associated with significantly lower rates of CVD in the general population and in subjects with CKD. ➢ The adjusted odd of CVD for those with light wine consumption was 0.72 (CI 0.52–0.99, *p* = 0.046) for the subjects with CKD.	[116]
**Green Tea and Coffee**			
6 healthy subjects and 54 hemodialysis patients	Three different doses (0, 455, and 910 mg) of oral catechins vs. oral vitamin C (500 mg)	➢ Reduced hemodialysis-enhanced plasma hypochlorous acid activity. ➢ Significantly reduced proinflammatory cytokine expression enhanced by hemodialysis. ➢ Lower pre-dialysis plasma hydrogen peroxide activity, lower hypochlorous acid activity, and lower phosphatidylcholine hydroperoxide, C-reactive protein, and pro-inflammatory cytokine concentrations.	[125]
40 patients with CKD requiring chronic dialysis	Catechin group: green tea (5 g/day for 1 month). Control group: water	➢ No significant change in clinical characteristics, oxidative stress, inflammatory markers, and circulating EPCs number. ➢ Significantly improved FMD in the catechin group (from 5.68 ± 2.67% to 8.66 ± 3.46%, *p* = 0.002). Short-term green tea consumption induced a rapid improvement in FMD, but did not improve circulating EPC levels in patients with CKD.	[126]
8717 subjects with normal renal function	0 coffee per week, <1 cup per week, 1–6 cups per week, 1 cup per day, and ≥2 cups per day	➢ Rates of decline in glomerular filtration were lower in daily coffee consumers. ➢ 1 cup per day (HR, 0.76, 95% confidence interval, 0.63–0.92) and ≥2 cups per day (HR, 0.80, 95% confidence interval, 0.65–0.98) were associated with a lower risk of chronic kidney disease development vs. non-drinkers.	[128]
4722 participants from the Doetinchem Cohort Study	Coffee and tea consumption (in cups/day)	➢ Tea consumption was not associated with eGFR. ➢ Individuals who drank >6 cups coffee/day had a 1.33 (95% CI: 0.24, 2.43) mL/min/m^2^ higher eGFR than those who drank <1 cup/day (P-trend = 0.02).	[129]
**Vitamin D Supplementation**			
Randomized, double-blind, placebo-controlled trial of 78 CKD subjects with iPTH >70 pg/mL and serum total 25-hydroxyvitamin D <30 ng/mL.	Daily treatment for six weeks with oral MR calcifediol (30, 60, or 90 µg) or a placebo	➢ Mean plasma iPTH decreased from baseline (140.3 pg/mL) by 20.9 ± 6.2% (SE), 32.8 ± 5.7, and 39.3 ± 4.3% in the 30, 60, and 90 µg of dose groups (*p* < 0.005). ➢ No clinically significant safety concerns arose during MR calcifediol treatment.	[138]
A systematic review and meta-analysis, 14 observational studies (194,932 patients)	Vitamin D compounds (25-hydroxyvitamin D, 1,25-dihydroxyvitamin D, and synthetic derivatives) vs. placebo	➢ Reduced risk of all-cause mortality (relative risk 0.73, 95%, CI 0.65–0.82). ➢ Risk reduction was greater in patients with higher parathyroid hormone serum levels (*p* = 0.01). ➢ Significantly reduced risk of cardiovascular mortality (relative risk 0.63, 95% CI 0.44–0.92). Therapies with 1,25-dihydroxyvitamin D and analogues are associated with reduced mortality in CKD patients, and, particularly, in those suffering from secondary hyperparathyroidism.	[139]
Randomized, double-blind, placebo-controlled trial, 120 patients with nondiabetic CKD stage 3–4 and vitamin D deficiency	Cholecalciferol (300,000 IU) or matching placebo	➢ Significantly increased endothelium-dependent brachial artery flow-mediated dilation at 16 weeks (mean change: 5.49%, 95% Cl 4.34% to 6.64%, *p* < 0.001). ➢ Significant favorable changes in pulse wave velocity and circulating IL-6 levels. In nondiabetic patients with stage 3–4 CKD and vitamin D deficiency, vitamin D supplementation may improve vascular function.	[144]
41 children with CKD and 24 healthy subjects with low 25-hydroxyvitamin D3 (25OHD) levels	Stoss vitamin D supplementation	➢ Significantly improved FMD and local arterial stiffness in patients. ➢ Homocysteine showed inverse correlation with baseline vitamin D level in CKD children and von Willebrand factor emerged as an independent risk factor for FMD impairment.	[145]
Multinational, double-blind, randomized placebo-controlled trial, 227 patients with CKD, mild-to-moderate LVH, and preserved LVEF	Oral paricalcitol, 2 μg/d (*n* = 115), or matching placebo (*n* = 112).	➢ Reduced parathyroid hormone levels within 4 weeks and maintained normal levels throughout the study duration. ➢ No differences in LV mass index and peak early diastolic lateral mitral annular tissue velocity (paricalcitol group, −0.01 cm/s [95% CI, −0.63 to 0.60 cm/s] vs. placebo group, −0.30 cm/s [95% CI, −0.93 to 0.34 cm/s]) Hypercalcemia were more frequent in the paricalcitol group when compared with the placebo group.	[143]
**Polyunsaturated Fatty Acids (PUFA)**			
Systematic review and meta-analysis of randomized controlled trials. Sixty trials (4129 participants).	n-3 PUFA supplementation	➢ Reduced cardiovascular death for participants on hemodialysis (RR) 0.45, 95% Cl 0.23–0.89), ➢ Prevention against ESRD (RR 0.30, CI 0.09–0.98) in CKD patients. ➢ No difference in all-cause mortality (RR 1.05, CI 0.84–1.33), acute transplant rejection (RR 0.98, CI 0.80–1.21), or allograft loss (RR 0.98, CI 0.54–1.81).	[156]
Analysis of longitudinal data from 1436 participants in the Diabetes Control and Complications Trial	Dietary n-3 long-chain polyunsaturated fatty acids (n-3 LC-PUFAs). Average intake of eicosapentaenoic and docosahexaenoic acid taken from diet histories.	➢ Decreased mean UAER (difference 22.7 mg/24 h [95% CI 1.6–43.8)]) in the top versus the bottom third of dietary n-3 LC-PUFAs. ➢ No association with incident albuminuria. ➢ Dietary n-3 LC-PUFAs appear inversely associated with the degree but not with the incidence of albuminuria in Type 1 diabetes.	[158]
Double-blind, permuted-block, randomized, placebo-controlled of ESRD patients	Fish-oil concentrate (study group) vs. corn-oil capsules (control group). Six 1-g soft-gel capsules each day for 6 months	➢ Significantly increased high-density lipoprotein cholesterol levels. ➢ Significantly increased LDL levels in both groups. The analysis indicates mixed results with respect to cardiovascular disease risk. Further research is required.	[160]
Double-blind randomized placebo-controlled design, 206 HD patients with documented CVD	n-3 PUFA or a control treatment (olive oil)	➢ Significantly higher serum phospholipid levels of n-3 PUFA in patients reporting high fish intake vs. low fish intake. ➢ Significant decrease in serum TG in n-3 PUFA vs. control group after three months (*p* = 0.01). ➢ No significant effect on TCh, HDL, LDL, Lp(a), or apoB.	[161]
12-month, prospective, single-blind, sequential intervention, cohort study of 24 HD patients	Three consecutive 4-month study periods taking the following supplements: A (olive oil: 2 g/day), B (omega-3 PUFA: 2 g/day), C (olive oil: 2 g/day).	➢ No significant impact on fibrinogen, hemoglobin, platelet, red, and white blood cell counts, PTH, PTT, serum TCh, TG, apo-A and B, CRP, and albumin levels. ➢ Significantly lower systolic (mean ± SD) (A: 131 ± 17.8 mm Hg, B: 122 ± 12.8 mm Hg, C: 129 ± 13.2 mm Hg), diastolic (A: 83 ± 16.3 mm Hg, B: 72 ± 14.8 mm Hg, C: 79 ± 6.5 mm Hg) and mean blood pressure (A: 99 ± 16.8 mm Hg, B: 88 ± 14.1 mm Hg, C: 96 ± 8.7 mm Hg) at the end of the study period B (*p* < 0.05). Omega-3 PUFA supplementation influenced only blood pressure in patients for long-term HD.	[162]
**Red Yeast Rice and Berberine**			
Forty moderately hypercholesterolemic outpatients with mild-to-moderate CKD and 40 cross-matched hypercholesterolemic subjects without CKD	Combined nutraceutical containing red yeast rice (3 mg monacolin K) and berberine (500 mg).	➢ No significant change in body mass index, blood pressure, liver transaminases, creatinine-phosphokinase, and eGFR. ➢ Improved TCh in non-CKD patients by (−21.6%), LDL-Cholesterol by (−24.2%), non HDL-Cholesterol (−24.0%), and TG (−20.8%). ➢ Improved TCh in CKD patients by (−21.1%), LDL-Cholesterol by (−23.7%), non HDL-Cholesterol (−23.9%), and TG (−20.4%). ➢ No difference among groups in terms of the effects on lipid metabolism. ➢ Significantly improved PWV in both groups (*p* < 0.01). No differences between groups.	[166]
Single centre, randomized, double-blind, placebo-controlled study, 50 hypercholesterolemic patients	Daily oral dose of NC (25 patients) or placebo, 6 weeks	➢ Decreased TCh (−1.14 ± 0.88 and −0.03 ± 0.78 mmol/L, *p* < 0.001), LDL (−1.06 ± 0.75 and −00.4 ± 0.54 mmol/L, *p* < 0.001) and TG levels. ➢ Improved endothelial-dependent flow-mediated dilation (3 ± 4% and 0 ± 3% respectively, *p* < 0.05) and insulin sensitivity in relation to NC. ➢ No adverse effect.	[168]
**Menaquinone-7 (MK-7)**			
42 non-dialyzed patients with CKD	Vitamin K2 at a dose of 90 μg (menaquinone, MK-7) + 10 μg of cholecalciferol (K + D group) or 10 μg of cholecalciferol (group D):	➢ Significantly lower increase in CCA-IMT n the K+D group vs. D group (ΔCCA-IMT, 0.06 ± 0.08 vs. 0.136 ± 0.05 mm, *p* = 0.005). ➢ Slightly lower increase in CACS in the K + D group vs. D group (ΔCACS, 58.1 ± 106.5 AU vs. 74.4 ± 127.1 AU, *p* = 0.7). ➢ Significant decrease in the level of dp-ucMGP and total OC in the K+D group. Vitamin K2 administration in patients with CKD stages 3-5 may limit the progression of atherosclerosis, but does not considerably affect the progression of calcification. Vitamin K2 significantly changes the levels of calcification promoters and inhibitors: dp-ucMGP, OC, and OPG.	[175]
Interventional randomized non-placebo-controlled trial with 3 parallel groups, 53 long-term hemodialysis patients in stable conditions	Menaquinone-7 (vitamin K(2)) treatment at 45, 135, or 360 μg/d for 6 weeks	➢ Dose-dependent and time-dependent decrease in circulating PIVKA-II and dephosphorylated-uncarboxylated MGP (response rates: 77% and 93% in the groups receiving 135 μg and 360 μg of menaquinone-7, respectively), uncarboxylated osteocalcin levels. Inactive MGP levels can be decreased markedly by daily vitamin K(2) supplementation.	[174]
Uremic rats	Control (high-phosphate diet), uremic (high-phosphate diet containing 0.5% adenine), and supplemented uremic diet (0.5% adenine, MK-7, magnesium carbonate, and Sucrosomial^®^ Iron), 6 weeks	➢ Supplemented uremic diet did not reduce creatinine, phosphate levels, or vascular calcification. ➢ Significant hypo-cholesterolemic effect (−18.9% in supplemental uremic vs. uremic diet, *p* < 0.05). New nutraceutical combination significantly impacts cholesterol metabolism and its supplementation may help to control mild hypercholesterolemic conditions in CKD patients.	[172]

CCA-IMT—carotid intima-media thickness. CETP—cholesteryl ester transfer protein. Cfd—complement factor D. CKD—chronic kidney disease. Cl—confidence interval. CMC—carboxymethylcellulose. CRP—C-reactive protein. CSS—chronic serum sickness. dp-ucMGP—desphosphorylated-uncarboxylated MGP. EDD—endothelium-dependent vasodilation. EF- ejection fraction. eGFR—estimated glomerular filtration rate. EPC - endothelial progenitor cells. ESRD – end-stage renal disease. FMD—flow-mediated dilatation. GPx—glutathione peroxidase. HD—maintenance hemodialysis. HR—hazard ratio. ICAM-1—Intercellular Adhesion Molecule 1. IL-6- interleukin-6. iPTH—plasma intact parathyroid hormone. LDL—low-density cholesterol. LVEF—left ventricular ejection fraction. LVH—left ventricular hypertrophy. LVSd—left ventricle dimension at the end-systole. MCP-1—monocyte chemoattractant protein-1. MK-7—menaquinone-7. MMP-2—metalloproteinase 2. OC—osteocalcin. OPG—osteoprotegerin. PTH—parathormone. PLA(2)—calcium-independent phospholipase A2. PPARgamma—peroxisome proliferator-activated receptor gamma. PTT—partial thromboplastin time. PWV - pulse wave velocity. RR- relative risk, SBP—systolic blood pressure. TGF- β—transforming growth factor-β. TNF-α—tumor necrosis factor-α. UAER -urinary albumin excretion rates. VCAM-1- Vascular cell adhesion protein 1.

## Data Availability

There is no supporting data.

## Abbreviations

ABCA1—ATP-binding cassette transporter; ACAT1—acetyl-CoA acetyltransferase 1; AKI—acute kidney injury; AMPK—5’AMP-activated protein kinase; aPWV—aortic pulse-wave velocity; BUN—blood urea nitrogen; CD36—cluster of differentiation 36; CKD—chronic kidney disease; CLA—conjugated linolenic acid; CVD—cardiovascular disease; CYP7A1—cholesterol 7 alpha-hydroxylase; DHA—docosahexaenoic acid; Dhcr24—24-Dehydrocholesterol Reductase; dp-ucMGP—desphosphorylated-uncarboxylated matrix Gla protein; ECM—extracellular matrix; EGCG—epigallocatechin-3-gallate; EPA—eicosapentaenoic acid; FGF15—fibroblast growth factor 15; FMD—flow-mediated dilatation; FXR—farnesoid X receptor; Gpx1 and Gpx4—glutathione peroxidase 1 and 4; GSH—glutathione; GST—glutathione S-transferase; Gstk1—glutathione S-Transferase Kappa 1; HD—hemodialysis; HDL—high-density lipoprotein; HF—high fat; HMG-CoA—β-hydroxy β-methylglutaryl-CoA; HO-1—heme oxygenase-1; HR—hazard ratio; IAP—intestinal alkaline phosphatase; ICAM-1—intercellular adhesion molecule 1; IL-1β—interleukin 1β; IL-6—interleukin-6; KLF2—Krüppel-like factor 2; LDL—low-density lipoprotein; Ldlr(-/-)—low-density lipoprotein receptor deficient; LF—low fat; LPS—lipopolysaccharide; LV—left ventricle; LXRα—liver X receptor α; MDA—malondialdehyde; MK-7—menaquinone-7; MMP-2—metalloproteinase 2; NADPH—nicotinamide adenine dinucleotide phosphate; NF-κB—nuclear factor kappa B; NHANES—National Health and Nutrition Examination Survey; NO—nitric oxide; NOS3—nitric oxide synthase; Nrf2—nuclear factor erythroid 2-related factor 2; Nx—nephrectomized; OC—osteocalcin; OPG—osteoprotegerin; PDGF—platelet derived growth factor; PDGF—platelet-derived growth factor; PIVKA-II—vitamin K absence II; PPAR-γ—peroxisome proliferator-activated receptor gamma γ; Prdx2—peroxiredoxin 2; PRIMO—Paricalcitol Capsule Benefits in Renal Failure-Induced Cardiac Morbidity; PTH—parathormon; PUFA—polyunsaturated fatty acids; PWV—pulse wave velocity; RCT—randomized clinical trial; ROS—reactive oxygen species; RSV—resveratrol; SHPT—secondary hyperparathyroidism; SIRT1—sirtuin 1; Sod2—superoxide dismutase 2; SR-B1—scavenger receptor, class B type 1; STZ—streptozotocin; TGFβ—transforming growth factor β; TMAO—trimethylamine-N-oxide; TNFα—tumor necrosis factor-α; Txnip—thioredoxin interacting protein; UUO—unilateral ureteral obstruction; VDR—vitamin D receptor; VEGF—vascular endothelial growth factor

## References

[1] Coresh J., Selvin E., Stevens L.A., Manzi J., Kusek J.W., Eggers P., Van Lente F., Levey A.S. (2007). Prevalence of chronic kidney disease in the United States. Jama.

[2] Keith D.S., Nichols G.A., Gullion C.M., Brown J.B., Smith D.H. (2004). Longitudinal follow-up and outcomes among a population with chronic kidney disease in a large managed care organization. Arch. Intern. Med..

[3] Ghosh S.S., Gehr T.W., Ghosh S. (2014). Curcumin and chronic kidney disease (CKD): Major mode of action through stimulating endogenous intestinal alkaline phosphatase. Molecules.

[4] Himmelfarb J., Stenvinkel P., Ikizler T.A., Hakim R.M. (2002). The elephant in uremia: Oxidant stress as a unifying concept of cardiovascular disease in uremia. Kidney Int..

[5] Moradi H., Sica D.A., Kalantar-Zadeh K. (2013). Cardiovascular burden associated with uremic toxins in patients with chronic kidney disease. Am. J. Nephrol..

[6] Zhu J., Du C. (2020). Could grape-based food supplements prevent the development of chronic kidney disease?. Crit. Rev. Food Sci. Nutr..

[7] Bergeron R., Previs S.F., Cline G.W., Perret P., Russell R.R., Young L.H., Shulman G.I. (2001). Effect of 5-aminoimidazole-4-carboxamide-1-beta-D-ribofuranoside infusion on in vivo glucose and lipid metabolism in lean and obese Zucker rats. Diabetes.

[8] Stenvinkel P. (2001). Inflammatory and atherosclerotic interactions in the depleted uremic patient. Blood Purif..

[9] Stenvinkel P., Ketteler M., Johnson R.J., Lindholm B., Pecoits-Filho R., Riella M., Heimbürger O., Cederholm T., Girndt M. (2005). IL-10, IL-6, and TNF-alpha: Central factors in the altered cytokine network of uremia--the good, the bad, and the ugly. Kidney Int..

[10] Choi B.H., Kang K.S., Kwak M.K. (2014). Effect of redox modulating NRF2 activators on chronic kidney disease. Molecules.

[11] Cybulsky A.V. (2000). Growth factor pathways in proliferative glomerulonephritis. Curr. Opin. Nephrol. Hypertens..

[12] Kruse N.T. (2019). Nutraceuticals as a potential adjunct therapy toward improving vascular health in CKD. Am. J. Physiol. Regul. Integr. Comp. Physiol..

[13] Ghiadoni L., Cupisti A., Huang Y., Mattei P., Cardinal H., Favilla S., Rindi P., Barsotti G., Taddei S., Salvetti A. (2004). Endothelial dysfunction and oxidative stress in chronic renal failure. J. Nephrol..

[14] Rossi S.H., McQuarrie E.P., Miller W.H., Mackenzie R.M., Dymott J.A., Moreno M.U., Taurino C., Miller A.M., Neisius U., Berg G.A. (2013). Impaired renal function impacts negatively on vascular stiffness in patients with coronary artery disease. BMC Nephrol..

[15] Cosola C., Sabatino A., di Bari I., Fiaccadori E., Gesualdo L. (2018). Nutrients, nutraceuticals, and xenobiotics affecting renal health. Nutrients.

[16] Ajay A.K., Vig S., Sabbisetti V. (2019). Mechanism of action of functional lipids and metabolites for patients with chronic kidney disease. Funct. Foods Health Dis..

[17] Sulaiman M.K. (2019). Diabetic nephropathy: Recent advances in pathophysiology and challenges in dietary management. Diabetol. Metab. Syndr..

[18] Mahmoodpoor F., Rahbar Saadat Y., Barzegari A., Ardalan M., Zununi Vahed S. (2017). The impact of gut microbiota on kidney function and pathogenesis. Biomed. Pharmacother..

[19] Sabatino A., Regolisti G., Cosola C., Gesualdo L., Fiaccadori E. (2017). Intestinal microbiota in type 2 diabetes and chronic kidney disease. Curr. Diab. Rep..

[20] Iriondo-DeHond A., Uribarri J., Castillo M., Ferranti P., Berry E.M., Anderson J.R. (2019). Usefulness of dietary components as sustainable nutraceuticals for chronic kidney disease. Encyclopedia of Food Security and Sustainability.

[21] Singh R., Geetanjali (2013). Nutraceuticals: Promising health product. Int. Res. J. Med. Sci..

[22] Santini A., Tenore G.C., Novellino E. (2017). Nutraceuticals: A paradigm of proactive medicine. Eur. J. Pharm. Sci..

[23] Bergamin A., Mantzioris E., Cross G., Deo P., Garg S., Hill A.M. (2019). Nutraceuticals: Reviewing their role in chronic disease prevention and management. Pharmaceut. Med..

[24] Salis S. (2018). Role of nutraceuticals and probiotics in chronic kidney disease. J. Renal. Nutr. Metab..

[25] Burnett A.J., Livingstone K.M., Woods J.L., McNaughton S.A. (2017). Dietary supplement use among Australian adults: Findings from the 2011–2012 national nutrition and physical activity survey. Nutrients.

[26] Jian Z. (2007). Nutraceuticals, nutritional therapy, phytonutrients, and phytotherapy for improvement of human health: A perspective on plant biotechnology application. Recent Pat. Biotechnol..

[27] Roberfroid M.B. (2000). Concepts and strategy of functional food science: The European perspective. Am. J. Clin. Nutr..

[28] German J.B., Walzem R.L. (2000). The health benefits of wine. Ann. Rev. Nutr..

[29] Găman M.-A., Epingeac M., Diaconu C., Găman A. (2020). Evaluation of oxidative stress levels in obesity and diabetes by the free oxygen radical test and free oxygen radical defence assays and correlations with anthropometric and laboratory parameters. World J. Diabet..

[30] Rossman M.J., LaRocca T.J., Martens C.R., Seals D.R. (2018). Healthy lifestyle-based approaches for successful vascular aging. J. Appl. Physiol..

[31] Al-Okbi S.Y., Mohamed D.A., Hamed T.E., Esmail R., Donya S.M. (2014). Prevention of renal dysfunction by nutraceuticals prepared from oil rich plant foods. Asian Pac. J. Trop. Biomed..

[32] Costa R.M., Magalhães A.S., Pereira J.A., Andrade P.B., Valentão P., Carvalho M., Silva B.M. (2009). Evaluation of free radical-scavenging and antihemolytic activities of quince (*Cydonia oblonga*) leaf: A comparative study with green tea (*Camellia sinensis*). Food Chem. Toxicol..

[33] Yang J., Liu R.H., Halim L. (2009). Antioxidant and antiproliferative activities of common edible nut seeds. LWT Food Sci. Technol..

[34] Dreher M.L. (2012). Pistachio nuts: Composition and potential health benefits. Nutr. Rev..

[35] Wall R., Ross R.P., Fitzgerald G.F., Stanton C. (2010). Fatty acids from fish: The anti-inflammatory potential of long-chain omega-3 fatty acids. Nutr. Rev..

[36] Almomen S.M., Guan Q., Liang P., Yang K., Sidiqi A.M., Levin A., Du C. (2017). Daily intake of grape powder prevents the progression of kidney disease in obese type 2 diabetic ZSF1 rats. Nutrients.

[37] Luciano R.L. (2014). Acute kidney injury from cherry concentrate in a patient with CKD. Am. J. Kidney Dis..

[38] Cory H., Passarelli S., Szeto J., Tamez M., Mattei J. (2018). The role of polyphenols in human health and food systems: A mini-review. Front. Nutr..

[39] Vauzour D., Rodriguez-Mateos A., Corona G., Oruna-Concha M.J., Spencer J.P. (2010). Polyphenols and human health: Prevention of disease and mechanisms of action. Nutrients.

[40] Chuengsamarn S., Rattanamongkolgul S., Phonrat B., Tungtrongchitr R., Jirawatnotai S. (2014). Reduction of atherogenic risk in patients with type 2 diabetes by curcuminoid extract: A randomized controlled trial. J. Nutr. Biochem..

[41] Akazawa N., Choi Y., Miyaki A., Tanabe Y., Sugawara J., Ajisaka R., Maeda S. (2012). Curcumin ingestion and exercise training improve vascular endothelial function in postmenopausal women. Nutr. Res..

[42] Draijer R., de Graaf Y., Slettenaar M., de Groot E., Wright C.I. (2015). Consumption of a polyphenol-rich grape-wine extract lowers ambulatory blood pressure in mildly hypertensive subjects. Nutrients.

[43] Nardini M., Natella F., Scaccini C. (2007). Role of dietary polyphenols in platelet aggregation. A review of the supplementation studies. Platelets.

[44] Forman H.J., Davies K.J., Ursini F. (2014). How do nutritional antioxidants really work: Nucleophilic tone and para-hormesis versus free radical scavenging in vivo. Free Radic. Biol. Med..

[45] Reuland D.J., McCord J.M., Hamilton K.L. (2013). The role of Nrf2 in the attenuation of cardiovascular disease. Exerc. Sport Sci. Rev..

[46] Scapagnini G., Vasto S., Abraham N.G., Caruso C., Zella D., Fabio G. (2011). Modulation of Nrf2/ARE pathway by food polyphenols: A nutritional neuroprotective strategy for cognitive and neurodegenerative disorders. Mol. Neurobiol..

[47] Goszcz K., Duthie G.G., Stewart D., Leslie S.J., Megson I.L. (2017). Bioactive polyphenols and cardiovascular disease: Chemical antagonists, pharmacological agents or xenobiotics that drive an adaptive response?. Br. J. Pharmacol..

[48] Cicero A.F.G., Caliceti C., Fogacci F., Giovannini M., Calabria D., Colletti A., Veronesi M., Roda A., Borghi C. (2017). Effect of apple polyphenols on vascular oxidative stress and endothelium function: A translational study. Mol. Nutr. Food Res..

[49] Roumeliotis S., Roumeliotis A., Dounousi E., Eleftheriadis T., Liakopoulos V. (2019). Dietary antioxidant supplements and uric acid in chronic kidney disease: A review. Nutrients.

[50] Gliozzi M., Malara N., Muscoli S., Mollace V. (2016). The treatment of hyperuricemia. Int. J. Cardiol..

[51] Mafra D., Borges N.A., Lindholm B., Shiels P.G., Evenepoel P., Stenvinkel P. (2020). Food as medicine: Targeting the uraemic phenotype in chronic kidney disease. Nat. Rev. Nephrol..

[52] Fasano A. (2011). Zonulin and its regulation of intestinal barrier function: The biological door to inflammation, autoimmunity, and cancer. Physiol. Rev..

[53] Deng Q., Liang L., Liu Q., Duan W., Jiang Y., Zhang L. (2018). Autophagy is a major mechanism for the dual effects of curcumin on renal cell carcinoma cells. Eur. J. Pharmacol..

[54] Metzler M., Pfeiffer E., Schulz S.I., Dempe J.S. (2013). Curcumin uptake and metabolism. Biofactors.

[55] Gupta S.C., Patchva S., Aggarwal B.B. (2013). Therapeutic roles of curcumin: Lessons learned from clinical trials. AAPS J..

[56] Hatcher H., Planalp R., Cho J., Torti F.M., Torti S.V. (2008). Curcumin: From ancient medicine to current clinical trials. Cell Mol. Life Sci..

[57] Aggarwal B.B. (2010). Targeting inflammation-induced obesity and metabolic diseases by curcumin and other nutraceuticals. Annu. Rev. Nutr..

[58] Prasad S., Gupta S.C., Tyagi A.K., Aggarwal B.B. (2014). Curcumin, a component of golden spice: From bedside to bench and back. Biotechnol. Adv..

[59] Hernández-Reséndiz S., Correa F., García-Niño W.R., Buelna-Chontal M., Roldán F.J., Ramírez-Camacho I., Delgado-Toral C., Carbó R., Pedraza-Chaverrí J., Tapia E. (2015). Cardioprotection by curcumin post-treatment in rats with established chronic kidney disease. Cardiovasc. Drugs Ther..

[60] Jiménez-Osorio A.S., García-Niño W.R., González-Reyes S., Álvarez-Mejía A.E., Guerra-León S., Salazar-Segovia J., Falcón I., Montes de Oca-Solano H., Madero M., Pedraza-Chaverri J. (2016). The effect of dietary supplementation with curcumin on redox status and nrf2 activation in patients with nondiabetic or diabetic proteinuric chronic kidney disease: A pilot study. J. Ren. Nutr..

[61] Shoskes D., Lapierre C., Cruz-Correa M., Muruve N., Rosario R., Fromkin B., Braun M., Copley J. (2005). Beneficial effects of the bioflavonoids curcumin and quercetin on early function in cadaveric renal transplantation: A randomized placebo controlled trial. Transplantation.

[62] Fan Y., Chen H., Peng H., Huang F., Zhong J., Zhou J. (2017). Molecular mechanisms of curcumin renoprotection in experimental acute renal injury. Front. Pharmacol..

[63] Sun P.P., Perianayagam M.C., Jaber B.L. (2009). Endotoxin-binding affinity of sevelamer: A potential novel anti-inflammatory mechanism. Kidney Int. Suppl..

[64] Bentala H., Verweij W.R., Huizinga-Van der Vlag A., van Loenen-Weemaes A.M., Meijer D.K., Poelstra K. (2002). Removal of phosphate from lipid A as a strategy to detoxify lipopolysaccharide. Shock.

[65] Ghosh S.S., Salloum F.N., Abbate A., Krieg R., Sica D.A., Gehr T.W., Kukreja R.C. (2010). Curcumin prevents cardiac remodeling secondary to chronic renal failure through deactivation of hypertrophic signaling in rats. Am. J. Physiol. Heart Circ. Physiol..

[66] Ghosh S.S., Krieg R., Massey H.D., Sica D.A., Fakhry I., Ghosh S., Gehr T.W. (2012). Curcumin and enalapril ameliorate renal failure by antagonizing inflammation in 5/6 nephrectomized rats: Role of phospholipase and cyclooxygenase. Am. J. Physiol. Renal. Physiol..

[67] Aparicio-Trejo O.E., Tapia E., Molina-Jijón E., Medina-Campos O.N., Macías-Ruvalcaba N.A., León-Contreras J.C., Hernández-Pando R., García-Arroyo F.E., Cristóbal M., Sánchez-Lozada L.G. (2017). Curcumin prevents mitochondrial dynamics disturbances in early 5/6 nephrectomy: Relation to oxidative stress and mitochondrial bioenergetics. Biofactors.

[68] Jacob A., Chaves L., Eadon M.T., Chang A., Quigg R.J., Alexander J.J. (2013). Curcumin alleviates immune-complex-mediated glomerulonephritis in factor-H-deficient mice. Immunology.

[69] Moreillon J.J., Bowden R.G., Deike E., Griggs J., Wilson R., Shelmadine B., Cooke M., Beaujean A. (2013). The use of an anti-inflammatory supplement in patients with chronic kidney disease. J. Complement. Integr. Med..

[70] Ye J. (2008). Regulation of PPARgamma function by TNF-alpha. Biochem. Biophys. Res. Commun..

[71] Ghosh S.S., Massey H.D., Krieg R., Fazelbhoy Z.A., Ghosh S., Sica D.A., Fakhry I., Gehr T.W. (2009). Curcumin ameliorates renal failure in 5/6 nephrectomized rats: Role of inflammation. Am. J. Physiol. Renal. Physiol..

[72] Shin S.K., Ha T.Y., McGregor R.A., Choi M.S. (2011). Long-term curcumin administration protects against atherosclerosis via hepatic regulation of lipoprotein cholesterol metabolism. Mol. Nutr. Food Res..

[73] Liu T., Li C., Sun H., Luo T., Tan Y., Tian D., Guo Z. (2014). Curcumin inhibits monocyte chemoattractant protein-1 expression and enhances cholesterol efflux by suppressing the c-Jun N-terminal kinase pathway in macrophage. Inflamm. Res..

[74] Hasan S.T., Zingg J.M., Kwan P., Noble T., Smith D., Meydani M. (2014). Curcumin modulation of high fat diet-induced atherosclerosis and steatohepatosis in LDL receptor deficient mice. Atherosclerosis.

[75] Ghosh S.S., Bie J., Wang J., Ghosh S. (2014). Oral supplementation with non-absorbable antibiotics or curcumin attenuates western diet-induced atherosclerosis and glucose intolerance in LDLR-/- mice--role of intestinal permeability and macrophage activation. PLoS ONE.

[76] Al-Rubaei Z.M., Mohammad T.U., Ali L.K. (2014). Effects of local curcumin on oxidative stress and total antioxidant capacity in vivo study. Pak. J. Biol. Sci..

[77] Kon V., Linton M.F., Fazio S. (2011). Atherosclerosis in chronic kidney disease: The role of macrophages. Nat. Rev. Nephrol..

[78] Khajehdehi P., Pakfetrat M., Javidnia K., Azad F., Malekmakan L., Nasab M.H., Dehghanzadeh G. (2011). Oral supplementation of turmeric attenuates proteinuria, transforming growth factor-β and interleukin-8 levels in patients with overt type 2 diabetic nephropathy: A randomized, double-blind and placebo-controlled study. Scand. J. Urol. Nephrol..

[79] Aggarwal B.B., Sung B. (2009). Pharmacological basis for the role of curcumin in chronic diseases: An age-old spice with modern targets. Trends Pharmacol. Sci..

[80] Maccarrone M., Taccone-Gallucci M., Finazzi-Agrò A. (2003). 5-Lipoxygenase-mediated mitochondrial damage and apoptosis of mononuclear cells in ESRD patients. Kidney Int. Suppl..

[81] Liakopoulos V., Roumeliotis S., Bozikas A., Eleftheriadis T., Dounousi E. (2019). Antioxidant supplementation in renal replacement therapy patients: Is there evidence?. Oxid. Med. Cell Longev..

[82] Jhun J., Min H.-K., Na H.S., Kwon J.y., Ryu J., Cho K.-H., Choi J., Jung K., Lee S.-Y., Kim S.J. (2020). Combinatmarion treatment with Lactobacillus acidophilus LA-1, vitamin B, and curcumin ameliorates the progression of osteoarthritis by inhibiting the pro-inflammatory mediators. Immunol. Lett..

[83] Buhrmann C., Mobasheri A., Matis U., Shakibaei M. (2010). Curcumin mediated suppression of nuclear factor-κB promotes chondrogenic differentiation of mesenchymal stem cells in a high-density co-culture microenvironment. Arthritis Res. Ther..

[84] Todhunter P.G., Kincaid S.A., Todhunter R.J., Kammermann J.R., Johnstone B., Baird A.N., Hanson R.R., Wright J.M., Lin H.C., Purohit R.C. (1996). Immunohistochemical analysis of an equine model of synovitis-induced arthritis. Am. J. Vet. Res..

[85] Shakibaei M., John T., Schulze-Tanzil G., Lehmann I., Mobasheri A. (2007). Suppression of NF-kappaB activation by curcumin leads to inhibition of expression of cyclo-oxygenase-2 and matrix metalloproteinase-9 in human articular chondrocytes: Implications for the treatment of osteoarthritis. Biochem. Pharmacol..

[86] Shakibaei M., Schulze-Tanzil G., John T., Mobasheri A. (2005). Curcumin protects human chondrocytes from IL-l1beta-induced inhibition of collagen type II and beta1-integrin expression and activation of caspase-3: An immunomorphological study. Ann. Anat..

[87] Gaedeke J., Noble N.A., Border W.A. (2004). Curcumin blocks multiple sites of the TGF-beta signaling cascade in renal cells. Kidney Int..

[88] Hu Y., Liang H., Du Y., Zhu Y., Wang X. (2010). Curcumin inhibits transforming growth factor-beta activity via inhibition of Smad signaling in HK-2 cells. Am. J. Nephrol..

[89] Yang Q., Wu S., Mao X., Wang W., Tai H. (2013). Inhibition effect of curcumin on TNF-α and MMP-13 expression induced by advanced glycation end products in chondrocytes. Pharmacology.

[90] Wang J., Wang X., Cao Y., Huang T., Song D.X., Tao H.R. (2018). Therapeutic potential of hyaluronic acid/chitosan nanoparticles for the delivery of curcuminoid in knee osteoarthritis and an in vitro evaluation in chondrocytes. Int. J. Mol. Med..

[91] Zhou L., Hu Y., Li C., Yan Y., Ao L., Yu B., Fang W., Liu J., Li Y. (2018). Levo-corydalmine alleviates vincristine-induced neuropathic pain in mice by inhibiting an NF-kappa B-dependent CXCL1/CXCR2 signaling pathway. Neuropharmacology.

[92] Pandey K.B., Rizvi S.I. (2009). Plant polyphenols as dietary antioxidants in human health and disease. Oxid. Med. Cell. Longev..

[93] Csiszar A., Smith K., Labinskyy N., Orosz Z., Rivera A., Ungvari Z. (2006). Resveratrol attenuates TNF-α-induced activation of coronary arterial endothelial cells: Role of NF-κB inhibition. Am. J. Physiol. Heart Circ. Physiol..

[94] Li J.Y., Huang W.Q., Tu R.H., Zhong G.Q., Luo B.B., He Y. (2020). Retraction Note: Resveratrol rescues hyperglycemia-induced endothelial dysfunction via activation of Akt. Acta Pharmacol. Sin..

[95] Yousuf S., Atif F., Ahmad M., Hoda N., Ishrat T., Khan B., Islam F. (2009). Resveratrol exerts its neuroprotective effect by modulating mitochondrial dysfunctions and associated cell death during cerebral ischemia. Brain Res..

[96] Sato M., Ray P.S., Maulik G., Maulik N., Engelman R.M., Bertelli A.A., Bertelli A., Das D.K. (2000). Myocardial protection with red wine extract. J. Cardiovasc. Pharmacol..

[97] Wallerath T., Deckert G., Ternes T., Anderson H., Li H., Witte K., Förstermann U. (2002). Resveratrol, a polyphenolic phytoalexin present in red wine, enhances expression and activity of endothelial nitric oxide synthase. Circulation.

[98] Xia N., Daiber A., Förstermann U., Li H. (2017). Antioxidant effects of resveratrol in the cardiovascular system. Br. J. Pharmacol..

[99] Xia N., Strand S., Schlufter F., Siuda D., Reifenberg G., Kleinert H., Förstermann U., Li H. (2013). Role of SIRT1 and FOXO factors in eNOS transcriptional activation by resveratrol. Nitric. Oxide.

[100] Wood J.G., Rogina B., Lavu S., Howitz K., Helfand S.L., Tatar M., Sinclair D. (2004). Sirtuin activators mimic caloric restriction and delay ageing in metazoans. Nature.

[101] Valenzano D.R., Terzibasi E., Genade T., Cattaneo A., Domenici L., Cellerino A. (2006). Resveratrol prolongs lifespan and retards the onset of age-related markers in a short-lived vertebrate. Curr. Biol..

[102] Howitz K.T., Bitterman K.J., Cohen H.Y., Lamming D.W., Lavu S., Wood J.G., Zipkin R.E., Chung P., Kisielewski A., Zhang L.L. (2003). Small molecule activators of sirtuins extend Saccharomyces cerevisiae lifespan. Nature.

[103] Xia N., Daiber A., Habermeier A., Closs E.I., Thum T., Spanier G., Lu Q., Oelze M., Torzewski M., Lackner K.J. (2010). Resveratrol reverses endothelial nitric-oxide synthase uncoupling in apolipoprotein E knockout mice. J. Pharmacol. Exp. Ther..

[104] Ferrero M.E., Bertelli A.E., Fulgenzi A., Pellegatta F., Corsi M.M., Bonfrate M., Ferrara F., de Caterina R., Giovannini L., Bertelli A. (1998). Activity in vitro of resveratrol on granulocyte and monocyte adhesion to endothelium. Am. J. Clin. Nutr..

[105] Leikert J.F., Räthel T.R., Wohlfart P., Cheynier V., Vollmar A.M., Dirsch V.M. (2002). Red wine polyphenols enhance endothelial nitric oxide synthase expression and subsequent nitric oxide release from endothelial cells. Circulation.

[106] Förstermann U., Li H. (2011). Therapeutic effect of enhancing endothelial nitric oxide synthase (eNOS) expression and preventing eNOS uncoupling. Br. J. Pharmacol..

[107] Gracia-Sancho J., Villarreal G., Zhang Y., García-Cardeña G. (2010). Activation of SIRT1 by resveratrol induces KLF2 expression conferring an endothelial vasoprotective phenotype. Cardiovasc. Res..

[108] Hammad A.S.A., Ahmed A.F., Heeba G.H., Taye A. (2019). Heme oxygenase-1 contributes to the protective effect of resveratrol against endothelial dysfunction in STZ-induced diabetes in rats. Life Sci..

[109] Chen M.L., Yi L., Zhang Y., Zhou X., Ran L., Yang J., Zhu J.D., Zhang Q.Y., Mi M.T. (2016). Resveratrol attenuates trimethylamine-N-oxide (TMAO)-induced atherosclerosis by regulating TMAO synthesis and bile acid metabolism via remodeling of the gut microbiota. mBio.

[110] Wellman A.S., Metukuri M.R., Kazgan N., Xu X., Xu Q., Ren N.S.X., Czopik A., Shanahan M.T., Kang A., Chen W. (2017). Intestinal epithelial sirtuin 1 regulates intestinal inflammation during aging in mice by altering the intestinal microbiota. Gastroenterology.

[111] Buhrmann C., Busch F., Shayan P., Shakibaei M. (2014). Sirtuin-1 (SIRT1) is required for promoting chondrogenic differentiation of mesenchymal stem cells. J. Biol. Chem..

[112] Li W., Cai L., Zhang Y., Cui L., Shen G. (2015). Intra-articular resveratrol injection prevents osteoarthritis progression in a mouse model by activating SIRT1 and thereby silencing HIF-2alpha. J. Orthop. Res..

[113] Dvir-Ginzberg M., Mobasheri A., Kumar A. (2016). The role of sirtuins in cartilage homeostasis and osteoarthritis. Curr. Rheumatol. Rep..

[114] Buhrmann C., Popper B., Aggarwal B.B., Shakibaei M. (2017). Resveratrol downregulates inflammatory pathway activated by lymphotoxin α (TNF-β) in articular chondrocytes: Comparison with TNF-α. PLoS ONE.

[115] Limagne E., Lançon A., Delmas D., Cherkaoui-Malki M., Latruffe N. (2016). Resveratrol interferes with IL1-β-induced pro-inflammatory paracrine interaction between primary chondrocytes and macrophages. Nutrients.

[116] Jespersen T., Kruse N., Mehta T., Kuwabara M., Noureddine L., Jalal D. (2018). Light wine consumption is associated with a lower odd for cardiovascular disease in chronic kidney disease. Nutr. Metab. Cardiovasc. Dis..

[117] Lewandowska U., Szewczyk K., Hrabec E., Janecka A., Gorlach S. (2013). Overview of metabolism and bioavailability enhancement of polyphenols. J. Agric. Food Chem..

[118] Csaki C., Mobasheri A., Shakibaei M. (2009). Synergistic chondroprotective effects of curcumin and resveratrol in human articular chondrocytes: Inhibition of IL-1beta-induced NF-kappaB-mediated inflammation and apoptosis. Arthritis Res. Ther..

[119] Lecumberri E., Dupertuis Y.M., Miralbell R., Pichard C. (2013). Green tea polyphenol epigallocatechin-3-gallate (EGCG) as adjuvant in cancer therapy. Clin. Nutr..

[120] Riegsecker S., Wiczynski D., Kaplan M.J., Ahmed S. (2013). Potential benefits of green tea polyphenol EGCG in the prevention and treatment of vascular inflammation in rheumatoid arthritis. Life Sci..

[121] Steinmann J., Buer J., Pietschmann T., Steinmann E. (2013). Anti-infective properties of epigallocatechin-3-gallate (EGCG), a component of green tea. Br. J. Pharmacol..

[122] Chen W.C., Hayakawa S., Shimizu K., Chien C.T., Lai M.K. (2004). Catechins prevents substance P-induced hyperactive bladder in rats via the downregulation of ICAM and ROS. Neurosci. Lett..

[123] Wang Y., Wang B., Du F., Su X., Sun G., Zhou G., Bian X., Liu N. (2015). Epigallocatechin-3-gallate attenuates oxidative stress and inflammation in obstructive nephropathy via NF-κB and Nrf2/HO-1 signalling pathway regulation. Basic Clin. Pharmacol. Toxicol..

[124] Wongmekiat O., Peerapanyasut W., Kobroob A. (2018). Catechin supplementation prevents kidney damage in rats repeatedly exposed to cadmium through mitochondrial protection. Naunyn. Schmiedebergs Arch. Pharmacol..

[125] Hsu S.P., Wu M.S., Yang C.C., Huang K.C., Liou S.Y., Hsu S.M., Chien C.T. (2007). Chronic green tea extract supplementation reduces hemodialysis-enhanced production of hydrogen peroxide and hypochlorous acid, atherosclerotic factors, and proinflammatory cytokines. Am. J. Clin. Nutr..

[126] Park C.S., Kim W., Woo J.S., Ha S.J., Kang W.Y., Hwang S.H., Park Y.W., Kim Y.S., Ahn Y.K., Jeong M.H. (2010). Green tea consumption improves endothelial function but not circulating endothelial progenitor cells in patients with chronic renal failure. Int. J. Cardiol..

[127] Lin Y., Shi D., Su B., Wei J., Găman M.-A., Sedanur Macit M., Borges do Nascimento I.J., Guimaraes N.S. (2020). The effect of green tea supplementation on obesity: A systematic review and dose–response meta-analysis of randomized controlled trials. Phytother. Res..

[128] Jhee J.H., Nam K.H., An S.Y., Cha M.U., Lee M., Park S., Kim H., Yun H.R., Kee Y.K., Park J.T. (2018). Effects of coffee intake on incident chronic kidney disease: A community-based prospective cohort study. Am. J. Med..

[129] Herber-Gast G.C., van Essen H., Verschuren W.M., Stehouwer C.D., Gansevoort R.T., Bakker S.J., Spijkerman A.M. (2016). Coffee and tea consumption in relation to estimated glomerular filtration rate: Results from the population-based longitudinal doetinchem cohort study. Am. J. Clin. Nutr..

[130] Tofovic S.P., Jackson E.K. (1999). Effects of long-term caffeine consumption on renal function in spontaneously hypertensive heart failure prone rats. J. Cardiovasc. Pharmacol..

[131] Holick M.F. (2007). Vitamin D Deficiency. N. Eng. J. Med..

[132] Dusso A.S. (2011). Kidney disease and vitamin D levels: 25-hydroxyvitamin D, 1,25-dihydroxyvitamin D., and VDR activation. Kidney Int. Suppl..

[133] Caravaca-Fontán F., Gonzales-Candia B., Luna E., Caravaca F. (2016). Relative importance of the determinants of serum levels of 25-hydroxy vitamin D in patients with chronic kidney disease. Nefrología.

[134] Cardoso M.P., Pereira L.A.L. (2019). Native vitamin D in pre-dialysis chronic kidney disease. Nefrología.

[135] Navaneethan S.D., Schold J.D., Arrigain S., Jolly S.E., Jain A., Schreiber M.J., Simon J.F., Srinivas T.R., Nally J.V. (2011). Low 25-hydroxyvitamin d levels and mortality in non–dialysis-dependent CKD. Am. J. Kidney Dis..

[136] Ravani P., Malberti F., Tripepi G., Pecchini P., Cutrupi S., Pizzini P., Mallamaci F., Zoccali C. (2009). Vitamin D levels and patient outcome in chronic kidney disease. Kidney Int..

[137] (2017). Kidney Disease: Improving Global Outcomes (KDIGO) CKD-MBD update work group. KDIGO 2017 clinical practice guideline update for the diagnosis, evaluation, prevention, and treatment of chronic kidney disease-mineral and bone disorder (CKD-MBD). Kidney Int. Suppl..

[138] Sprague S.M., Silva A.L., Al-Saghir F., Damle R., Tabash S.P., Petkovich M., Messner E.J., White J.A., Melnick J.Z., Bishop C.W. (2014). Modified-release calcifediol effectively controls secondary hyperparathyroidism associated with vitamin D insufficiency in chronic kidney disease. Am. J. Nephrol..

[139] Sprague S.M., Crawford P.W., Melnick J.Z., Strugnell S.A., Ali S., Mangoo-Karim R., Lee S., Petkovich P.M., Bishop C.W. (2016). Use of extended-release calcifediol to treat secondary hyperparathyroidism in stages 3 and 4 chronic kidney disease. Am. J. Nephrol..

[140] Pilz S., Iodice S., Zittermann A., Grant W.B., Gandini S. (2011). Vitamin D status and mortality risk in CKD: A meta-analysis of prospective studies. Am. J. Kidney Dis..

[141] Căpuşa C., Stefan G., Stancu S., Ilyes A., Dorobanţu N., Mircescu G. (2016). Subclinical cardiovascular disease markers and vitamin D deficiency in non-dialysis chronic kidney disease patients. Arch. Med. Sci..

[142] Duranton F., Rodriguez-Ortiz M.E., Duny Y., Rodriguez M., Daurès J.P., Argilés A. (2013). Vitamin D treatment and mortality in chronic kidney disease: A systematic review and meta-analysis. Am. J. Nephrol..

[143] Selamet U., Katz R., Ginsberg C., Rifkin D.E., Fried L.F., Kritchevsky S.B., Hoofnagle A.N., Bibbins-Domingo K., Drew D., Harris T. (2018). Serum calcitriol concentrations and kidney function decline, heart failure, and mortality in elderly community-living adults: The health, aging, and body composition study. Am. J. Kidney Dis..

[144] Kumar V., Yadav A.K., Lal A., Kumar V., Singhal M., Billot L., Gupta K.L., Banerjee D., Jha V. (2017). A Randomized trial of vitamin d supplementation on vascular function in CKD. J. Am. Soc. Nephrol..

[145] Aytaç M.B., Deveci M., Bek K., Kayabey Ö., Ekinci Z. (2016). Effect of cholecalciferol on local arterial stiffness and endothelial dysfunction in children with chronic kidney disease. Pediatr. Nephrol..

[146] Thadhani R., Appelbaum E., Pritchett Y., Chang Y., Wenger J., Tamez H., Bhan I., Agarwal R., Zoccali C., Wanner C. (2012). Vitamin D therapy and cardiac structure and function in patients with chronic kidney disease: The PRIMO randomized controlled trial. Jama.

[147] Wang A.Y., Fang F., Chan J., Wen Y.Y., Qing S., Chan I.H., Lo G., Lai K.N., Lo W.K., Lam C.W. (2014). Effect of paricalcitol on left ventricular mass and function in CKD--the OPERA trial. J. Am. Soc. Nephrol..

[148] Levin G.P., Robinson-Cohen C., de Boer I.H., Houston D.K., Lohman K., Liu Y., Kritchevsky S.B., Cauley J.A., Tanaka T., Ferrucci L. (2012). Genetic variants and associations of 25-hydroxyvitamin D concentrations with major clinical outcomes. Jama.

[149] Jean G., Souberbielle J.C., Chazot C. (2017). Vitamin D in chronic kidney disease and dialysis patients. Nutrients.

[150] Navaneethan S.D., Virani S.S. (2017). Omega-3 Fatty Acids (Fish Oil) supplementation and albuminuria: Not a slam dunk. J. Am. Heart Assoc..

[151] Wakimoto T., Kondo H., Nii H., Kimura K., Egami Y., Oka Y., Yoshida M., Kida E., Ye Y., Akahoshi S. (2011). Furan fatty acid as an anti-inflammatory component from the green-lipped mussel *Perna canaliculus*. Proc. Natl. Acad. Sci. USA.

[152] Calder P.C., Grimble R.F. (2002). Polyunsaturated fatty acids, inflammation and immunity. Eur. J. Clin. Nutr..

[153] Aguila M.B., Pinheiro A.R., Aquino J.C., Gomes A.P., Mandarim-de-Lacerda C.A. (2005). Different edible oil beneficial effects (canola oil, fish oil, palm oil, olive oil, and soybean oil) on spontaneously hypertensive rat glomerular enlargement and glomeruli number. Prostaglandins Other Lipid Mediat..

[154] Yokoyama M., Tanigawa K., Murata T., Kobayashi Y., Tada E., Suzuki I., Nakabou Y., Kuwahata M., Kido Y. (2010). Dietary polyunsaturated fatty acids slow the progression of diabetic nephropathy in streptozotocin-induced diabetic rats. Nutr. Res..

[155] Zanetti M., Gortan Cappellari G., Barbetta D., Semolic A., Barazzoni R. (2017). Omega 3 polyunsaturated fatty acids improve endothelial dysfunction in chronic renal failure: Role of eNOS activation and of oxidative stress. Nutrients.

[156] Saglimbene V.M., Wong G., van Zwieten A., Palmer S.C., Ruospo M., Natale P., Campbell K., Teixeira-Pinto A., Craig J.C., Strippoli G.F.M. (2020). Effects of omega-3 polyunsaturated fatty acid intake in patients with chronic kidney disease: Systematic review and meta-analysis of randomized controlled trials. Clin. Nutr..

[157] Baggio B., Musacchio E., Priante G. (2005). Polyunsaturated fatty acids and renal fibrosis: Pathophysiologic link and potential clinical implications. J. Nephrol..

[158] Lee C.C., Sharp S.J., Wexler D.J., Adler A.I. (2010). Dietary intake of eicosapentaenoic and docosahexaenoic acid and diabetic nephropathy: Cohort analysis of the diabetes control and complications trial. Diabetes Care.

[159] Matsushita K., van der Velde M., Astor B.C., Woodward M., Levey A.S., de Jong P.E., Coresh J., Gansevoort R.T. (2010). Association of estimated glomerular filtration rate and albuminuria with all-cause and cardiovascular mortality in general population cohorts: A collaborative meta-analysis. Lancet.

[160] Bowden R.G., Jitomir J., Wilson R.L., Gentile M. (2009). Effects of omega-3 fatty acid supplementation on lipid levels in end-stage renal disease patients. J. Ren. Nutr..

[161] Svensson M., Schmidt E.B., Jørgensen K.A., Christensen J.H. (2008). The effect of n-3 fatty acids on lipids and lipoproteins in patients treated with chronic haemodialysis: A randomized placebo-controlled intervention study. Nephrol. Dial Transplant..

[162] Vernaglione L., Cristofano C., Chimienti S. (2008). Omega-3 polyunsaturated fatty acids and proxies of cardiovascular disease in hemodialysis: A prospective cohort study. J. Nephrol..

[163] Ogawa J., Kishino S., Ando A., Sugimoto S., Mihara K., Shimizu S. (2005). Production of conjugated fatty acids by lactic acid bacteria. J. Biosci. Bioeng..

[164] Yuan G., Sinclair A.J., Xu C., Li D. (2009). Incorporation and metabolism of punicic acid in healthy young humans. Mol. Nutr. Food Res..

[165] Ogborn M.R., Nitschmann E., Bankovic-Calic N., Weiler H.A., Fitzpatrick-Wong S., Aukema H.M. (2003). Dietary conjugated linoleic acid reduces PGE_2_ release and interstitial injury in rat polycystic kidney disease. Kid. Int..

[166] Cicero A. (2013). Effect of a lipid-lowering nutraceutical on pulse-wave-velocity in hypercholesterolemic patients with or without chronic kidney disease. Open Hypertens. J..

[167] Cicero A.F., Tartagni E., Borghi C. (2012). Nutraceuticals with lipid-lowering activity: Do they have any effect beyond cholesterol reduction?. Clin. Lipidol..

[168] Affuso F., Ruvolo A., Micillo F., Saccà L., Fazio S. (2010). Effects of a nutraceutical combination (berberine, red yeast rice and policosanols) on lipid levels and endothelial function randomized, double-blind, placebo-controlled study. Nutr. Metab. Cardiovasc. Dis..

[169] Asbaghi O., Ghanbari N., shekari M., Reiner Ž., Amirani E., Hallajzadeh J., Mirsafaei L., Asemi Z. (2020). The effect of berberine supplementation on obesity parameters, inflammation and liver function enzymes: A systematic review and meta-analysis of randomized controlled trials. Clin. Nutr. ESPEN.

[170] Fusaro M., Crepaldi G., Maggi S., Galli F., D’Angelo A., Calò L., Giannini S., Miozzo D., Gallieni M. (2010). Vitamin K, bone fractures, and vascular calcifications in chronic kidney disease: An important but poorly studied relationship. J. Endocrinol. Investig..

[171] Yasufumi N., Yusuke H., Fumiaki I., Toshio K. (1990). Effect of simvastatin (MK-733) on the regulation of cholesterol synthesis in Hep G2 cells. Biochem. Pharmacol..

[172] Lupo M.G., Biancorosso N., Brilli E., Tarantino G., Adorni M.P., Vivian G., Salvalaio M., Dall’Acqua S., Sut S., Neutel C. (2020). Cholesterol-lowering action of a novel nutraceutical combination in uremic rats: Insights into the molecular mechanism in a hepatoma cell line. Nutrients.

[173] Geleijnse J.M., Vermeer C., Grobbee D.E., Schurgers L.J., Knapen M.H., van der Meer I.M., Hofman A., Witteman J.C. (2004). Dietary intake of menaquinone is associated with a reduced risk of coronary heart disease: The Rotterdam Study. J. Nutr..

[174] Keyzer C.A., Vermeer C., Joosten M.M., Knapen M.H., Drummen N.E., Navis G., Bakker S.J., de Borst M.H. (2015). Vitamin K status and mortality after kidney transplantation: A cohort study. Am. J. Kidney Dis..

[175] Kurnatowska I., Grzelak P., Masajtis-Zagajewska A., Kaczmarska M., Stefańczyk L., Vermeer C., Maresz K., Nowicki M. (2015). Effect of vitamin K2 on progression of atherosclerosis and vascular calcification in nondialyzed patients with chronic kidney disease stages 3–5. Pol. Arch. Med. Wewn..

[176] Cozzolino M., Mangano M., Galassi A., Ciceri P., Messa P., Nigwekar S. (2019). Vitamin K in chronic kidney disease. Nutrients.

[177] Westenfeld R., Krueger T., Schlieper G., Cranenburg E.C., Magdeleyns E.J., Heidenreich S., Holzmann S., Vermeer C., Jahnen-Dechent W., Ketteler M. (2012). Effect of vitamin K2 supplementation on functional vitamin K deficiency in hemodialysis patients: A randomized trial. Am. J. Kidney Dis..

[178] Cicero A.F., Ferroni A., Ertek S. (2012). Tolerability and safety of commonly used dietary supplements and nutraceuticals with lipid-lowering effects. Expert Opin. Drug Saf..

[179] Leena M.M., Silvia M.G., Vinitha K., Moses J.A., Anandharamakrishnan C. (2020). Synergistic potential of nutraceuticals: Mechanisms and prospects for futuristic medicine. Food Funct..

[180] Efferth T., Koch E. (2011). Complex interactions between phytochemicals. The multi-target therapeutic concept of phytotherapy. Curr. Drug. Targets.

[181] Rather M.A., Bhat B.A., Qurishi M.A. (2013). Multicomponent phytotherapeutic approach gaining momentum: Is the “one drug to fit all” model breaking down?. Phytomedicine.

[182] Cruz-Correa M., Shoskes D.A., Sanchez P., Zhao R., Hylind L.M., Wexner S.D., Giardiello F.M. (2006). Combination treatment with curcumin and quercetin of adenomas in familial adenomatous polyposis. Clin. Gastroenterol. Hepatol..

[183] Cai T., Mazzoli S., Bechi A., Addonisio P., Mondaini N., Pagliai R.C., Bartoletti R. (2009). Serenoa repens associated with Urtica dioica (ProstaMEV) and curcumin and quercitin (FlogMEV) extracts are able to improve the efficacy of prulifloxacin in bacterial prostatitis patients: Results from a prospective randomised study. Int. J. Antimicrob. Agents.

[184] Helal N.A., Eassa H.A., Amer A.M., Eltokhy M.A., Edafiogho I., Nounou M.I. (2019). Nutraceuticals’ novel formulations: The good, the bad, the unknown and patents involved. Recent Pat. Drug Deliv. Formul..

[185] Nounou M.I., Ko Y., Helal N.A., Boltz J.F. (2018). Adulteration and counterfeiting of online nutraceutical formulations in the United States: Time for intervention?. J. Diet Suppl..

[186] Asghar A., Randhawa M.A., Masood M.M., Abdullah M., Irshad M.A., Grumezescu A.M., Holban A.M. (2018). Chapter 10-nutraceutical formulation strategies to enhance the bioavailability and efficiency: An overview. Role of Materials Science in Food Bioengineering.

[187] Schmitt J., Ferro A. (2013). Nutraceuticals: Is there good science behind the hype?. Br. J. Clin. Pharmacol..

[188] Gil F., Hernández A.F., Martín-Domingo M.C., Gupta R.C. (2016). Chapter 58-toxic contamination of nutraceuticals and food ingredients. Nutraceuticals.

[189] Shaito A., Posadino A.M., Younes N., Hasan H., Halabi S., Alhababi D., Al-Mohannadi A., Abdel-Rahman W.M., Eid A.H., Nasrallah G.K. (2020). Potential adverse effects of resveratrol: A literature review. Int. J. Mol. Sci..

[190] NKF Use of Herbal Supplements in Chronic Kidney Disease. https://kidneyhi.org/use-of-herbal-supplements-in-chronic-kidney-disease.

[191] Yu J., Zhou Z., Tay-Sontheimer J., Levy R.H., Ragueneau-Majlessi I. (2017). Intestinal drug interactions mediated by OATPs: A systematic review of preclinical and clinical findings. J. Pharm. Sci..

[192] Diamond B.J., Bailey M.R. (2013). Ginkgo biloba: Indications, mechanisms, and safety. Psychiatr. Clin. N. Am..

[193] Rider C.V., Nyska A., Cora M.C., Kissling G.E., Smith C., Travlos G.S., Hejtmancik M.R., Fomby L.M., Colleton C.A., Ryan M.J. (2014). Toxicity and carcinogenicity studies of Ginkgo biloba extract in rat and mouse: Liver, thyroid, and nose are targets. Toxicol. Pathol..

[194] Shimizu M., Shirakami Y., Sakai H., Kubota M., Kochi T., Ideta T., Miyazaki T., Moriwaki H. (2015). Chemopreventive potential of green tea catechins in hepatocellular carcinoma. Int. J. Mol. Sci..

[195] Gupta R.C., Srivastava A., Lall R. (2018). Toxicity potential of nutraceuticals. Methods Mol. Biol..

[196] Levy I., Attias S., Ben-Arye E., Goldstein L., Schiff E. (2017). Adverse events associated with interactions with dietary and herbal supplements among inpatients. Br. J. Clin. Pharmacol..

[197] Mouly S., Lloret-Linares C., Sellier P.O., Sene D., Bergmann J.F. (2017). Is the clinical relevance of drug-food and drug-herb interactions limited to grapefruit juice and Saint-John’s Wort?. Pharmacol. Res..

[198] de Boer I.H., Caramori M.L., Chan J.C.N., Heerspink H.J.L., Hurst C., Khunti K., Liew A., Michos E.D., Navaneethan S.D., Olowu W.A. (2020). KDIGO 2020 clinical practice guideline for diabetes management in chronic kidney disease. Kidney Int..

[199] Levin A., Stevens P.E. (2014). Summary of KDIGO 2012 CKD guideline: Behind the scenes, need for guidance, and a framework for moving forward. Kidney Int..

